# A multi-crystal method for extracting obscured crystallographic states from conventionally uninterpretable electron density

**DOI:** 10.1038/ncomms15123

**Published:** 2017-04-24

**Authors:** Nicholas M. Pearce, Tobias Krojer, Anthony R. Bradley, Patrick Collins, Radosław P. Nowak, Romain Talon, Brian D. Marsden, Sebastian Kelm, Jiye Shi, Charlotte M. Deane, Frank von Delft

**Affiliations:** 1 Structural Genomics Consortium, Nuffield Department of Medicine, University of Oxford, Roosevelt Drive, Oxford OX3 7DQ, UK; 2 Diamond Light Source Ltd, Harwell Science and Innovation Campus, Didcot OX11 0QX, UK; 3 Kennedy Institute of Rheumatology, Nuffield Department of Orthopaedics, Rheumatology and Musculoskeletal Sciences, University of Oxford, Roosevelt Drive, Oxford OX3 7FY, UK; 4 UCB Pharma, 208 Bath Road, Slough SL1 3WE, UK; 5 Department of Statistics, University of Oxford, 24-29 St Giles, Oxford OX1 3LB, UK; 6 Department of Biochemistry, University of Johannesburg, Auckland Park 2006, South Africa

## Abstract

In macromolecular crystallography, the rigorous detection of changed states (for example, ligand binding) is difficult unless signal is strong. Ambiguous (‘weak' or ‘noisy') density is experimentally common, since molecular states are generally only fractionally present in the crystal. Existing methodologies focus on generating maximally accurate maps whereby minor states become discernible; in practice, such map interpretation is disappointingly subjective, time-consuming and methodologically unsound. Here we report the PanDDA method, which automatically reveals clear electron density for the changed state—even from inaccurate maps—by subtracting a proportion of the confounding ‘ground state'; changed states are objectively identified from statistical analysis of density distributions. The method is completely general, implying new best practice for all changed-state studies, including the routine collection of multiple ground-state crystals. More generally, these results demonstrate: the incompleteness of atomic models; that single data sets contain insufficient information to model them fully; and that accuracy requires further map-deconvolution approaches.

## Abstract (web summary)

Building a ligand into a weak region of an electron density map of a protein is a subjective process. Here, the authors present a new method to obtain a clear electron density for a bound ligand based on multi-crystal experiments and 3D background correction.

Besides its use for resolving the overall three-dimensional (3D) structure of bio-molecules, macromolecular X-ray crystallography (MX) is deployed extensively to observe small changes to known structures, especially compound binding in ligand-discovery and -development projects. Arriving at the final model once initial electron density estimates are available (after ‘phasing'), relies on a long-established and rarely questioned paradigm: cycling between building atoms into the current density estimate and computationally optimizing the model against the measured data (‘refinement'). The latter improves the calculated phases and yields more detailed density that should reveal additional model omissions and errors; the process is assumed to converge on a model that fully describes the crystal's content.

In practice, convergence is never convincingly achieved. Much density both strong and weak invariably remains unexplained (‘noisy'), hence the aphorism that ‘refinement […] is never finished, only abandoned'^1^, and hence too the ‘R-factor gap'^2^, which has obdurately resisted all methodology advances. More recent work has shown that conventional single-conformation models are too simplistic to describe the crystal^3^,^4^,^5^; and that electron density features far weaker than the conventional cut-off reflect model deficiencies rather than measurement error^6^,^7^.

Evidently then, near convergence, conventionally calculated (sigmaA-weighted^8^) density derived from a single data set is necessary but insufficient to complete the model, as it shows a superposition of states that is currently impossible to de-convolute algorithmically. Nearly complete models with discrete yet uninterpretable superpositions are common in systematic studies of perturbations involving few atoms, such as ligand binding, photochemical changes or radiation damage. Since even strong biophysical effects are contingent on crystal packing or integrity, only a subset of the crystal may transition away from the ground state, even after extensive optimization of the experiment. Finally, all current modelling approaches ultimately rely on shape matching, and density superpositions are susceptible to interpretation errors and bias^9^,^10^,^11^ (such as the problem of the ‘Ligand of Desire'^9^).

Existing methods to auto-generate multi-conformer models^4^,^5^ are not relevant when changes are chemical, and moreover have had little take-up, presumably because neither is explicit modelling involved nor have robust validation criteria emerged to allay long-cultivated fears of over-fitting^12^. Approaches from time-resolved crystallography^13^ apply only to specialized experiments.

In this work, we show that unencumbered views of the changed, non-ground state can be obtained by recasting the problem as a multi-data set, 3D background-correction problem (Fig. 1), which allows the relevant signal to be extracted from conventional single-data set density. An accurate estimate of the background can be obtained by averaging near-convergence density, in real space and after local alignment, from dozens (>30) of independently measured but approximately identical ground-state crystals. Subtraction of a suitable fraction of this background estimate from the near-convergence density of a data set containing a putative changed state, yields a residual partial-difference map that we call an event map, which is in general fully interpretable:


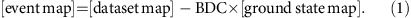


Identifying the optimal Background Density Correction factor (BDC) is essential for extracting the best signal, as illustrated schematically in Fig. 2, which also illustrates the problems with using conventional maps for the identification of minor crystallographic states.

## Results

### The PanDDA algorithm

Our new method—Pan-Dataset Density Analysis (PanDDA)—comprises: the characterization of a set of related crystallographic data sets of the same crystal form; the identification of (binding) events; and the subtraction of ground state density to reveal clear density for events.

The method builds on the principle of isomorphous difference (F_o_–F_o_) maps^14^, but analyses many maps simultaneously by first locally aligning maps in real space to bypass the requirement of strict isomorphism, and then directly comparing the best estimate of true electron density, namely sigmaA-weighted (2mF_o_–DF_c_) maps from late-stage refinement, which ensures that maps are correctly scaled.

Using multiple maps allows a *Z*-score measure to be calculated at each point in every data set, that reflects how significantly the data set deviates from the ensemble of data sets at that point in space. *Z*-scores are assembled into spatial *Z*-maps, and clusters of large *Z*-scores are an objective and statistically meaningful measure for potentially interesting crystallographic signal—events—such as a binding ligand. Using *Z*-maps addresses the common pitfall of over-interpreting density that is in fact ground state density, since in such cases, *Z*-scores will be small. Equally importantly, *Z*-maps also make it possible to identify weak changed states (for example, low-occupancy ligands) that do not yield strong difference (mF_o_–DF_c_) density.

Finally, the precise localization of each change enables reliable background subtraction at that site, where the optimal BDC is estimated as the value for which the ground state-subtracted map is locally least correlated to the ground-state map, relative to a normalizing global correlation across the unit cell (see Methods section). Using an averaged ground-state map for subtraction, as opposed to a single ground-state map, reduces experimental noise in the ground-state estimate and thereby also in the event map. Furthermore, averaging over multiple data sets minimizes the influence of stochastic variation between the data sets^15^ (characterized and discussed in Supplementary Methods). Finally, the averaging generates an estimate of the ground state that can be used directly as density, bypassing the need for any subjective modelling and map interpretation. The BDC is determined algorithmically and objectively, and results in event map density approximating only the changed configuration of the site, including protein backbone and side-chain conformations induced by the change.

### Application to crystallographic fragment screening data

We demonstrate the power of the method by applying it to the most demanding type of changed-state study, namely crystallographic fragment screening^16^,^17^, which attempts to observe in electron density the rare and often low occupancy binding events that occur when a relatively large (200–1,000) library of weak-binding ‘fragment' compounds (150–300 Da, 100 μM–10 mM)^18^,^19^ are added individually or as cocktails to a series of equivalent crystals. Conventionally, the analysis is challenging as it involves inspecting a lot of 3D space—the whole unit cell in each data set—for convincing evidence of bound fragments (‘hits'). In contrast, PanDDA directly eliminates the thousands of strong electron density blobs with no statistical significance, objectively identifying only regions that are unique to each data set; the ground-state data sets are provided by the many hit-free crystals.

**Figure 3:**
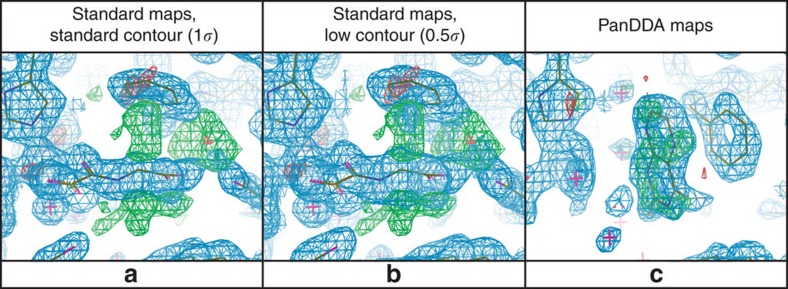
PanDDA maps clearly show detail obscured by conventional maps. JMJD2D fragment screening data set x401 at 1.48 Å. (**a**,**b**) Conventional maps (2mF_o_–DF_c_, blue, contour as indicated; mF_o_–DF_c_, green/red, ±3*σ*) are dominated by the NOG co-factor analogue bound in the majority fraction of the crystal, whereas (**c**) the event map (blue, 2*σ*, BDC=0.9) and the *Z*-map (green/red, *±*4) unambiguously reveal both ligand and associated changes in protein conformations.

**Figure 4:**
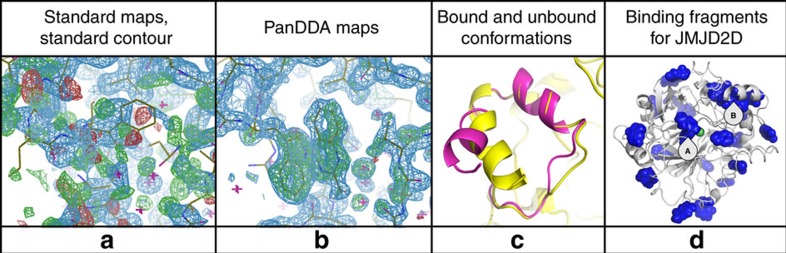
PanDDA maps reveal complex minor conformations and identify allosteric binders. In JMJD2D data set x402, at 1.45 Å, (**a**) conventional maps (contoured as in Fig. 3a) show a complex superposition of bound and unbound states that make it impossible to identify the bound state (the known unbound state is shown). (**b**) However, in PanDDA maps (contoured as in Fig. 3c, BDC=0.8) the bound conformation can be modelled easily (as shown). (**c**) Final models for the unbound (yellow) and bound (magenta) conformations highlight the large conformational change. (**d**) Fragments are detected to bind all over the surface of JMJD2D, revealing potential allosteric sites, including the peptide-binding groove (site A) and the large helix reordering (site B).

Applied to a series of fragment screens (Table 1), PanDDA yielded markedly more hits than manual inspection of density, far more quickly and all with high confidence (Figs 3 and 4; Supplementary Figs 1–6), in both known binding sites and new allosteric sites (Fig. 4d). Several fragments induced significant reordering of sections of the protein that could only be modelled with PanDDA event maps (Fig. 4a–c, Supplementary Fig. 1a–c), whilst also enabling the identification of mislabelled ligands and the discovery of experimental errors (Supplementary Figs 1d–f and 3d–f). Models erroneously built into misleading conventional density could be discarded with statistical confidence, and the binding of chemically elaborated hit compounds could be analysed more reliably. Full experimental details and complete descriptions are provided in Supplementary Note 1. The method also effectively disambiguates density in conventional ligand-binding studies with ligands co-crystallized and a sub-optimal number of ground-state data sets (Supplementary Note 2).

**Figure 5:**
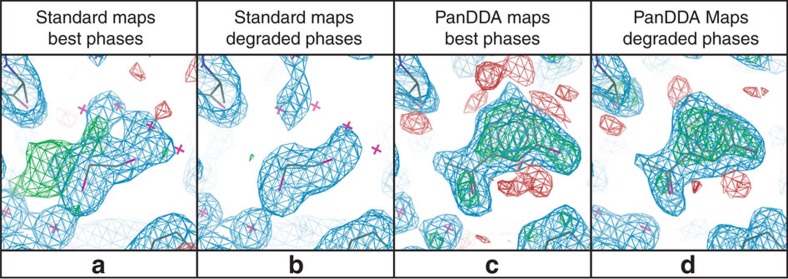
Weak ligand identification remains straightforward when phases are degraded. BAZ2B data sets were re-analysed using a deliberately sabotaged reference model, introducing a ∼30° phase error and increasing R_work_ and R_free_ by ∼12% for all dimple-refined data sets. Shown here is the weak hit (refined occupancy: 0.64) in data set x492, contoured for different maps as labelled: (**a**,**b**) 1.78 Å 2mF_o_–DF_c_ (blue, 1*σ*) and mF_o_–DF_c_ (green/red, ±3*σ*). (**c**,**d**) 1.79 Å event (blue, 2*σ*) and *Z*-maps (green/red, ±3). R_work_/R_free_ are 0.18/0.21 and 0.30/0.32 for best and degraded phases, respectively. BDCs for best and degraded phases are 0.77 and 0.73, respectively. Whereas with standard maps, degraded phases remove all evidence of an unmodelled change, in PanDDA maps, ligand identification is no more difficult, even if the quality of the density is predictably reduced.

Strikingly, detection of weak binding events is simple even when phases are far from convergence (Fig. 5).

### Model validation

Model validation is a long-established bedrock of crystallographic analysis^12^, and crucially requires a model that is numerically stable in refinement. As ligands—but especially fragments—invariably bind at sub-unitary occupancy, we generate an atomic ensemble model that reflects the crystal content implied by the density correction: the changed state modelled from event maps is combined with the ground-state model, with initial occupancy of the changed state set to 2*(1-BDC) (discussed in Supplementary Methods). Incorporation of the ground state into the model enforces our Bayesian prior knowledge of the crystal, that the ligand is most likely not bound to all copies of the protein in the crystal, and a superposition of the two states is thus the most likely situation. These ensemble models are indeed well-behaved in refinement, provided the ground state can be easily represented by an atomic model.

After refinement, some ligands built into strong event density would be considered invalid by comparison of the model and the refined density (Supplementary Fig. 7), or the subjective but best-practice criterion^9^ of visual assessment of agreement between model and conventional OMIT maps. As this is counterintuitive, given the clarity of the event maps, we instead formulated the following strong objective validation principles:

1. The changed-state partial model must conform to calculable numerical criteria (Table 2). We adopt established requirements: a strong correlation between the model and the observed density (real-space correlation coefficient, RSCC>0.7) and that ligand B-factors must be comparable to those of surrounding residues. We also apply a new metric, that modelling and refinement should result in negligible difference density around the site (real-space *Z*-difference score, RSZD<3)^20^, and further require that the model must not move under refinement (low heavy-atom root-mean-squared deviation before versus after). These metrics are fully defined in Methods and shown for all models in Supplementary Tables 1–4.
2. The ground state partial model is considered an immutable component of the crystal, with a status similar to common restraints (for example, geometry or non-crystallographic symmetry), as in general there is not enough diffraction information to propose otherwise. Thus, the ground state model needs to be fully complete before incorporation into the ensemble, and during further cycles of model building, it may not be altered, as it is a strong Bayesian prior. To stabilize refinement, it may need to be strongly restrained to the original ground state model (by external restraints using, for example, PROSMART^21^).
3. The primary event density must always be available when disseminating such models. All crystallographic data used for the PanDDA analysis must also be made available so that the analysis may be reproduced.

The group deposition feature recently added by the PDB^22^ makes it realistic to deposit all the many changed- and ground-state structures, as well as event maps (see Data Availability). On the other hand, standard mechanisms for presenting the validation evidence described above are yet to be finalized. Refinement programs do not yet support some external restraints that we predict will be important for numerical stability at low resolution or for very low occupancy at high resolution, in particular restraining relative B-factors to stabilize occupancy refinement; this is the subject of future work.

In general, only the changed state will be of primary scientific interest in the refined model, with the ground state essentially an experimental artefact. Unlike the artefacts inherent in any crystal structure, here they are explicitly declared and need not be inferred by further analysis. Structure repositories, whether public (PDB) or internal, would ideally support this by removing the ground state for normal use; this is only possible when states are logically labelled, as discussed in Methods.

## Discussion

The PanDDA algorithm fundamentally revises current methods through a more correct treatment of the crystallographic data, not only yielding dramatically improved signal-to-noise, but also providing rigorous measures of confidence in identified signal. This allows far more subtle changes to be modelled, whose importance will be experiment- and context-dependent: in ligand development, evidence of weak binding is now known to be productive for optimizing binding potency^23^. More generally, occupancy is subject to diffusion- and other solid-state effects inherent to the crystalline state, and will be an imperfect proxy for the scientific import of a change of interest. What matters most is that any changed state can be viewed as objectively and modelled as accurately as possible, which is what the PanDDA approach allows.

We thus propose a new standard practice for ligand binding and other changed-state studies, namely the collection of a series of ground state data sets before proceeding with the putative changed-state data sets, to provide the contrast necessary to identify the changes of interest.

Retrospective analysis indicates that ∼30 data sets are required for full convergence of the statistical model (Supplementary Methods), an experiment that can be completed within hours at modern synchrotron beamlines with fast pixel detectors^24^ and sample automation^25^, and that needs to be performed only once per crystal form. To address such an experiment's other bottleneck, the logistics of analysing large numbers of data sets, the PanDDA implementation includes graphical tools and various command-line options.

This number of data sets is required for identification of subtle changes from the ground state to be sensitive and robust, by ensuring that the *Z*-map represents a true statistical measure of changed-state signal. However, the background correction itself still works when fewer than 30 data sets are available (Supplementary Note 2), the trade-off being potentially reduced quality of the event maps. Future work will address whether the number of required data sets can be identified *a priori* for a given crystal system.

The PanDDA method is applicable and effective at any resolution, though at lower resolutions, as maps become less precise, higher occupancies of changed states will in general be required for them to be detected by *Z*-score. What matters most is the consistency of ground-state data sets so that they can be represented well by an average; therefore, in regions of crystals that vary considerably, such as crystal contacts, statistical confidence is reduced similarly to low resolutions.

As the algorithm currently uses a contrast-maximization approach to estimate BDC, event map density for changes appears somewhat stronger than density for unchanged atoms (typically, surrounding protein). In practice, this is not problematic, as the density for the changed states is generally clear, and unchanged conformations do not require modelling anyway. Establishing a BDC procedure that evens out this difference will require accounting for phase bias in the event maps, but falls outside the scope of this work.

In principle, the PanDDA approach will allow comparisons between different crystal forms of the same protein. However, since functionally important conformational changes are not only common in such cases but by their nature affect the functionally interesting regions, algorithmic treatment of the local alignment is complex and the topic of future work.

Our results upend a long-held tenet in macromolecular crystallographic model building, that to visualize subtle features requires optimal phase estimates and thus a model as complete and globally error-free as possible^26^. Conscientiously observed, this places a heavy time burden on the analysing scientist as it demands multiple iterations of modelling for each data set. The PanDDA approach makes this both practically and theoretically unnecessary: a single local modelling step fully validates an interpretation, even when the model retains minor problems elsewhere.

More generally, we submit that a qualitative shift in approaches to generating crystallographic models is now due. PanDDA addresses one class of experiments, those involving induced local changes, but all problems of uninterpretable density, and indeed some of the R-factor gap^2^, should be addressable by analogous map deconvolution methods. Multi-data set experiments are no longer difficult; nevertheless, existing tools for treating them focus on pursuing a single, representative data set through averaging^27^. Instead, what now appears key is to establish methods for targeted perturbations of poorly ordered regions, along with rigorous algorithms for reconstructing and visualizing discrete states, and for subsequent model validation.

## Methods

The PanDDA algorithm is schematically outlined in Supplementary Fig. 9 (Supplementary Methods).

### Data set preparation

The input to PanDDA is a series of refined crystallographic data sets, each consisting of a refined structure and associated diffraction data, including 2mF_o_–DF_c_ structure factors. These can come from any refinement program, as long as all data sets are refined using the same initial atomic model and the same protocol. All models of the protein must be identical, up to the numbering and labelling of atoms. All data sets used in this paper were prepared using the Dimple pipeline (part of CCP4 (ref. 28)), from reference models including solvent molecules; there is no requirement to remove solvent atoms from known binding sites.

### Structure and map alignment

To allow map voxels to be compared between crystals that are not exactly isomorphous, maps are aligned using the refined models as reference points.

The input protein structures are aligned using a flexible alignment algorithm (Supplementary Methods). Sections of the protein are aligned separately, to give alignment matrices for that section. The alignments generated from the structures are stored and are used to transform and thereby align the electron density maps.

### Handling variations of map resolutions

To allow map voxels to be compared between crystals, maps have to be calculated at the same level of detail, even though crystals can diffract to a wide range of resolutions. For analysing a specific data set, its full resolution is used; but for contributing to the analysis of a different data set, higher resolution data sets are truncated to the resolution of the target data set, while lower resolution data sets are ignored. Therefore, we analyse the collection of data sets at a number of resolutions, and high resolution data sets are used multiple times for characterization at lower resolutions, but will only be analysed once, at their highest possible resolution. Maps are recalculated using truncated diffraction data at each different resolution limit. Thus, if processing in resolution bins of 1.0, 1.5, 2, and 2.5 Å, a 1.2 Å data set would be analysed at 1.5 Å, but also be used to build distributions at 2 Å and 2.5 Å.

Fourier terms omitted in a given map, as happens when reflections are unobserved and then effectively set to zero, lead to systematic changes in electron density throughout the unit cell that strongly affect the outlier analysis; strong low-resolution terms are particularly problematic. Therefore, reflections in all data sets are truncated to the set of miller indices common to all data sets; and for map calculation, all missing Fourier terms are estimated as DF_c_, which refinement programs perform automatically as long as the indices are correctly included in the reflection files.

Truncated 2mF_o_–DF_c_ structure factors are Fourier-transformed to generate maps. These maps are aligned using the alignment transformations from the flexible alignment.

### Statistical model

Once maps for a particular resolution have been aligned, a statistical model is parameterized using the electron density of the ground-state data sets. The aligned maps are placed on an isotropic Cartesian grid, and the electron density is sampled at each grid point of each data set. The model treats the observed value of the electron density in data set *i*, at grid point *m*, as being sampled from a distribution


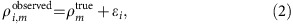


where 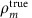 models the natural variation in the electron density at point *m*, independent of data set, and *ɛ*_*i*_ represents the experimental uncertainty in the electron density in data set *i*. The variability of the 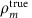
term accounts for the fact that the crystals are not identical, and that small local fluctuations may exist between the crystals. These areas are most likely to be in the crystal contacts, or flexible areas of the protein. 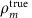
represents the ‘true' (unmeasurable) electron density for this crystal form, of which each crystal (and associated data set) is a sample.

The simplest model is to assume that both the uncertainty in electron density values as well as variation in electron density at a point arising from differences between the crystals, can be modelled by a normal distribution. Therefore, if


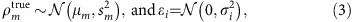


then


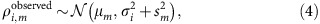


where *μ*_*m*_ is the mean value of the electron density at point *m*, *s*_*m*_ is the variance of the ‘true' electron density at point *m*, and *σ*_*i*_ is the uncertainty in data set *i*. Under this model, the parameters *μ*_*m*_ are estimated by taking the un-weighted average of all of the ground state densities.

The mean ground state map is used to estimate the data set uncertainty, *σ*_*i*_, for all data sets as follows. Subtracting the mean map from each data set map we obtain a mean difference map. By assuming that the experimental and model uncertainty in the electron density map are the major contributors to deviations from the mean map, the histogram of the mean-difference map values is used to estimate the total uncertainty of the data set. Calculating the quantiles of a theoretical normal distribution 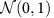 and plotting them against the quantiles from the mean-difference map, yields a Q-Q plot where the slope of the central portion of the map (between the ±1.5 theoretical quantiles) gives an estimate of the uncertainty of the data set (Supplementary Fig. 11a). This is equivalent to the method used in Tickle (2012) for calculating the uncertainty of an electron density map^20^.

To estimate *s*_*m*_, a maximum likelihood method is applied on our model in (4), using the observed values 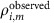, as well as estimates for *σ*_*i*_ and *μ*_*m*_ for the ground-state data sets (Supplementary Methods). An example comparison of the ‘raw' standard deviations of the grid points (simple s.d. of electron density values, not accounting for observation error) and the ‘adjusted' values is shown in Supplementary Fig. 12. This adjustment results in the majority of points having no variation that is not accounted for by the data set uncertainties; the remaining points have non-negligible variation, with non-zero *s*_*m*_, and these indicate naturally variable regions.

### Calculation of *Z*-maps

The parameterized statistical model allows the identification of areas of individual data set maps that deviate significantly from the mean map: ‘events'. Z-scores are calculated by


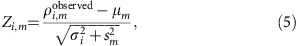


where large *Z*-scores indicate significant deviations from the mean map. The distributions of *Z*-scores for a particular data set have improved normality compared to the simple differences from the mean (Supplementary Fig. 11b), as expected.

Regions of individual data sets are identified as significant by contouring *Z*-maps at *Z*=2.5, and filtering remaining blobs by a minimum peak value of *Z*=3 and a minimum volume of 10 Å^3^ (volume of a water molecule is ∼30 Å^3^). Neighbouring blobs are grouped together if the minimum distance between them is <5 Å. These parameters were identified on the BAZ2B data set, and found appropriate in subsequent studies and are therefore the current program defaults.

### Calculation of event maps

For identified events, the background density correction (BDC) factor is estimated as follows. Different fractions of the mean map are subtracted from the data set map, and the correlation between the resulting map and the mean map is calculated both globally and for the area around the event, defined by the blob identified in the *Z*-map expanded by 1 Å.

Globally, the data set map looks similar to the mean map, so plotting the global correlation against the subtracted fraction yields a signal-to-noise curve, dropping off at a speed related to the noise in the data set (green dashed line, Supplementary Fig. 15). Locally to the identified site, however, the data set map is a superposition between something similar to the mean map and something that is unrelated (for example, density of bound ligand). As more of the mean map is subtracted, the local correlation between the mean map and the resulting map (black dashed line, Supplementary Fig. 15) will decrease faster than the global correlation. Subtracting the local correlation curve from the global correlation curve, BDC is estimated where the difference between these two correlation curves is maximized (blue solid line, Supplementary Fig. 15). The final event map is calculated as in equation (1).

### Model building and refinement

Interesting sites are identified by *Z*-maps and modelling is performed using a combination of *Z*-maps and event maps, similarly to the way that mF_o_–DF_c_ maps may be used to guide the modelling of 2mF_o_–DF_c_ maps. Modelling takes place in the aligned reference frame, as defined in Supplementary Methods.

After modelling of the changed state, the new conformations of the protein are merged with the ground state model. Atoms in the ground state that are not present or have moved in the changed state are assigned to a previously unused conformer (for example, C). Similarly, atoms in the changed state model that are not present in the ground state, or have moved, are assigned another unused conformer (for example, D). Atoms that are not changed between the two states remain unaltered. The resulting ensemble models are then back-transformed, using the flexible alignments, to the original crystallographic frame for refinement.

The models in Table 1 have then been refined as an ensemble using phenix.refine^29^,^30^, under conventional resolution-dependant refinement protocols, with constrained occupancy groups corresponding to the bound and unbound structures to ensure that the occupancies of the bound and unbound states sum to unity.

Because of the methodical way in which the ensembles are generated, the changed state model can be extracted simply by removing the atoms corresponding to the changed ground state atoms (that is, conformer C in the above example).

### Validation

The atomic model of the changed state is validated by four quality metrics (Table 2). Two are electron density scores, generated by *EDSTATS*^20^: real-space correlation coefficient (RSCC) reflects the fit of the atoms to the experimental density, and should typically be greater than 0.7; while real-space *Z*-difference score (RSZD) measures the amount of difference density that is found around these atoms, and should be below 3. The B-factor ratio measures the consistency of the model with surrounding protein, and is calculated from the B-factors of the changed atoms and all side-chain atoms within 4 Å, respectively. Large values (>3) reflect poor evidence for the model, and intermediate values (1.5+) indicate errors in refinement or modelling; for weakly-binding ligands, systematically large ratios may be justifiable. Coordinate root-mean-squared deviation (RMSD) compares the positions of all atoms built into event density, with their positions after final refinement, and should be below 1 Å.

### Implementation

PanDDA is implemented in Python and relies heavily on the CCTBX^31^. It has been tested extensively for robustness and usability by users of Diamond's XChem fragment screening facility. Source code is available on bitbucket (https://bitbucket.org/pandda/pandda) or as part of CCP4 (ref. 28). A manual and tutorial are available at https://pandda.bitbucket.io. Processing 200–500 data sets on a 3.7 GHz Quad-Core Intel Xeon with 32 GB of RAM takes ∼3–10+ hours depending on resolution binning and size of crystallographic unit cell.

### Data availability

Models were built and refined for those ligands that could be uniquely identified in the event maps, except for those that interact extensively with the crystal contacts and are therefore unlikely to be biologically relevant. Modelled data sets (those in Table 1) and unmodelled data sets have been deposited in the PDB using the new group deposition system (PDB codes for each data set are stated in Supplementary Table 5); structure factors for event maps are included within each mmCIF file downloadable from the PDB. PDB group deposition IDs for the ligand-bound structures are G_1002018 (BAZ2B), G_1002020 (JMJD2D), G_1002022 (BRD1), and G_1002024 (SP100); group IDs for the automatically refined structures are G_1002019 (BAZ2B), G_1002021 (JMJD2D), G_1002023 (BRD1), and G_1002025 (SP100). However, since navigation of this large numbers of structures and event maps remains an obstacle to interpretation, interactive summary pages^32^,^33^,^34^,^35^ for each fragment screen have been uploaded to Zenodo (https://zenodo.org); zip files of all of the crystallographic data have also been uploaded^36^,^37^,^38^,^39^ (Supplementary Table 5). All other data are available from the corresponding author upon reasonable request.

## Additional information

**How to cite this article:** Pearce, N. M. *et al*. A multi-crystal method for extracting obscured crystallographic states from conventionally uninterpretable electron density. *Nat. Commun.* **8,** 15123 doi: 10.1038/ncomms15123 (2017).

**Publisher's note**: Springer Nature remains neutral with regard to jurisdictional claims in published maps and institutional affiliations.

## Supplementary Material

Supplementary Information Supplementary figures, supplementary tables, supplementary notes and supplementary methods.

Peer Review File

## Figures and Tables

**Figure 1:**
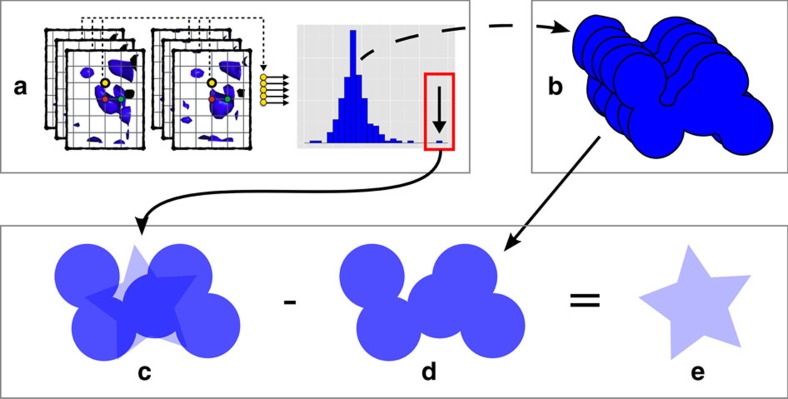
Schematic of how outlier identification and background subtraction reveals changed-state density. (**a**) Analysis of the aligned electron density distribution for the same voxel (yellow dots) identifies data sets which differ from the ensemble—outliers—such as those containing a bound ligand or other ‘changed state', for example the changed state in **c**. (**b**) Averaging over multiple ‘ground-state' data sets further provides an accurate estimate of the ground-state density, leading to **d**. With pixel intensity representing electron density strength, (**c**) shows an identified location, at which the density is a superposition of changed-state (20%) and ground-state (80%) densities; the changed state is obscured by the superposed ground state. (**d**) shows only the ground-state density, adjusted by applying a weighting (BDC=0.8). (**e**) The density that remains after subtracting the background yields an estimate of the changed state which is in general fully interpretable.

**Figure 2:**
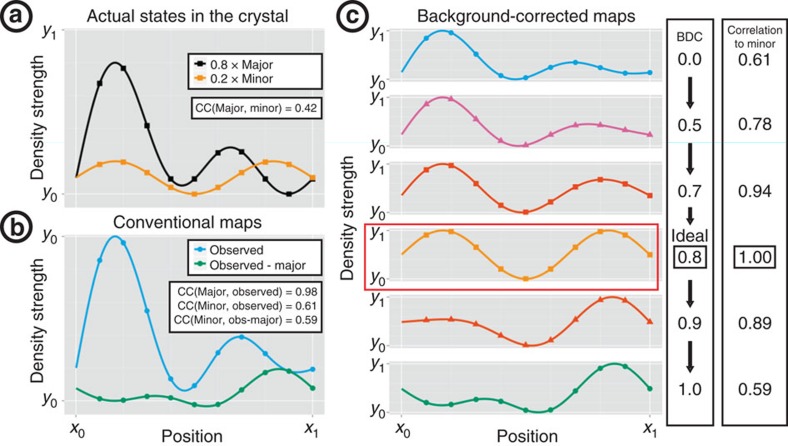
Minor conformations are obscured in conventional maps but are revealed by background correction. 1D simulations are used to illustrate 3D electron density. (**a**) The actual crystal contains 80% major (black) and 20% minor (orange) states, which are largely dissimilar (correlation: 0.42). (**b**) Conventional (2mF_o_–DF_c_) maps (blue) show only the superposition, which resembles the major far more than the interesting minor state (correlations: 0.98 and 0.59; in practice, the scale is arbitrary). Isomorphous difference (F_o_–F_o_) maps (green) show the subtraction of the full-occupancy major state from the observed data set, and do not resemble the minor state either, except where the major state has low density (right side). (**c**) ‘Event maps' (scaled for comparison), generated as in equation (1) for different values of BDC, reveal the minor state optimally for only one value of BDC (0.8, indicated in red). BDC=0.0 corresponds to the observed density, and BDC=1.0 to a F_o_–F_o_ map.

**Table 1:** Hit rates from fragment screens before and after use of PanDDA.

**Protein**	**JMJD2D**	**BAZ2B**	**SP100**	**BRD1**
Data sets	226	200	116	292
Resolution range (Å)	1.1–2.6	1.5–2.5	1.3–2.7	1.5–3.6
Identified hits (Human/**PanDDA**)	2/**24**	3/**9**	0/**2**	29/**40**
Identified hit rate (%) (Human/**PanDDA**)	0.9/**10.6**	1.5/**4.5**	0/**1.7**	9.9/**13.7**
Identified sites (Human/**PanDDA**)	1/**5**	1/**1**	0/**1**	1/**2**

PanDDA, Pan-Data set Density Analysis.

All fragment screens consisted of a single soaked compound per data set. An identified site comprises more than two binding ligands that are not heavily interacting with crystal contacts. Number of hits was determined as number of data sets containing a bound ligand. Hit rate was calculated as percentage of data sets containing bound ligands.

**Table 2:** Acceptable values of ligand validation scores.

**Metric**	**‘Good' range**
RSCC	>0.7
RSZD	<3
B-factor ratio	∼1
RMSD	<1

RMSD, Coordinate root-mean-squared deviation; RSCC, real-space correlation coefficient; RSZD, real-space *Z*-difference score. Scores are defined as in the Methods section.
