# Supplementary material for: A multi-crystal method for extracting obscured crystallographic states from conventionally uninterpretable electron density
Source: Nat Commun. 2017 Apr 24;8:15123. doi: 10.1038/ncomms15123 (PMC5413968; doi:10.1038/ncomms15123)
Supplement: Supplementary Information — Supplementary figures, supplementary tables, supplementary notes and supplementary methods. [file ncomms15123-s1.pdf]

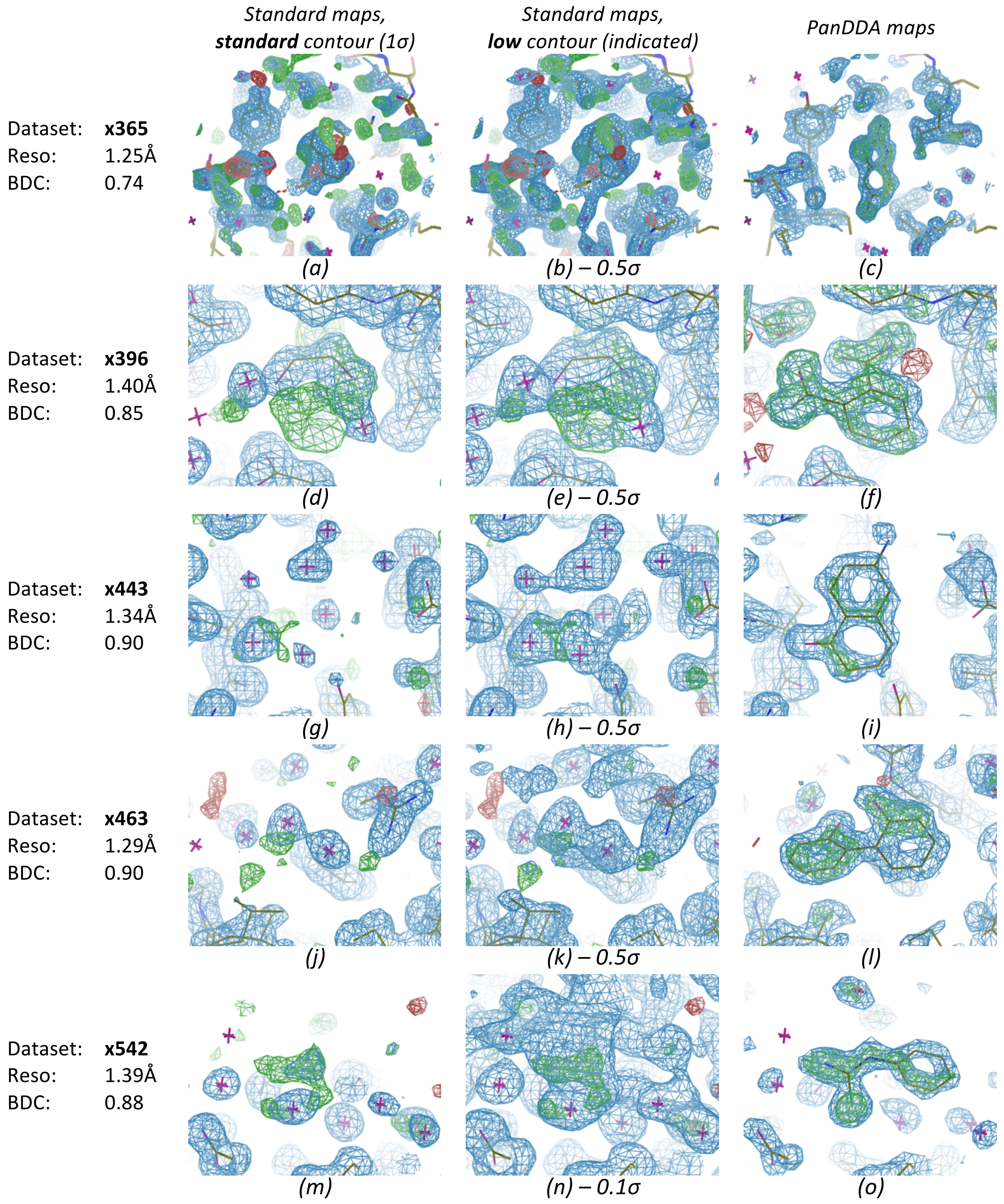

Supplementary Fig. 1. JMJD2D ligand examples. *Dataset statistics are as labelled in the first column; the first two columns show the* $2mF_o{-}DF_c$ *map (blue) contoured at the different levels indicated, and mFo−DFc map (green/red) contoured at ±3σ. The third column shows the PanDDA event map (blue) contoured at 2σ, and Z-Map (green/red) contoured at ±4.*

Dataset x365: The binding of the ligand induces a conformational change in the protein. Conventional maps show the density for both the reference conformation (shown) and the new ligand-induced conformation. This results in a complex superposition that is difficult to model. PanDDA maps show only density for the ligand-induced conformation, allowing it to be modelled easily.

Dataset x396: Binding and identity of the ligand are obscured by solvent in conventional maps, whereas PanDDA maps show clear density, which does not match the shape of the labelled ligand; the clarity of the event map enabled the identification of the modelled ligand by its chemical connectivity. The question of whether the bound ligand is disordered is also answered: the ligand is bound in one conformation only.

Datasets x443, x463 and x542: Conventional maps show evidence for nothing but solvent. PanDDA maps show clear density for a weakly bound ligand.

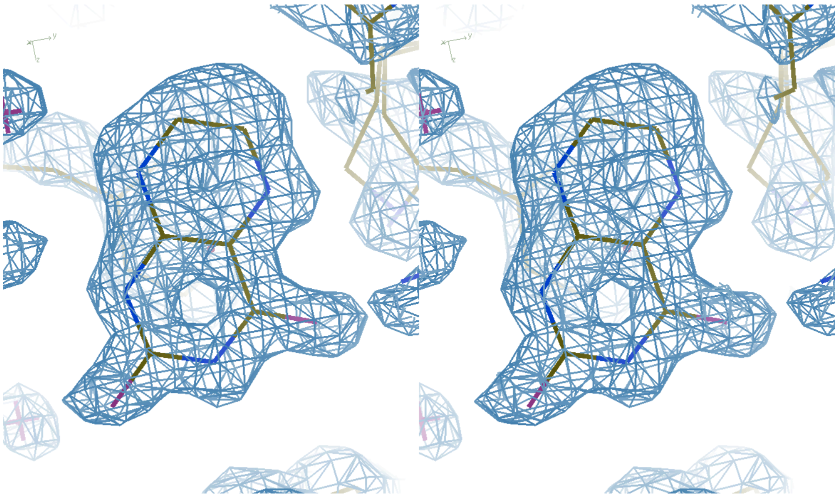

Supplementary Fig. 2. Stereo Image of the ligand in dataset JMJD2D-x401. *The event map (contoured at 2σ) shows clear evidence for the ligand (ligand as in Figure 3).*

	*Standard maps, **standard** contour (1σ)*	*Standard maps, **low** contour (indicated)*	*PanDDA maps*
Dataset: **x434**<br>Reso: 1.65Å<br>BDC: 0.74			
Dataset: **x538**<br>Reso: 1.77Å<br>BDC: 0.85			

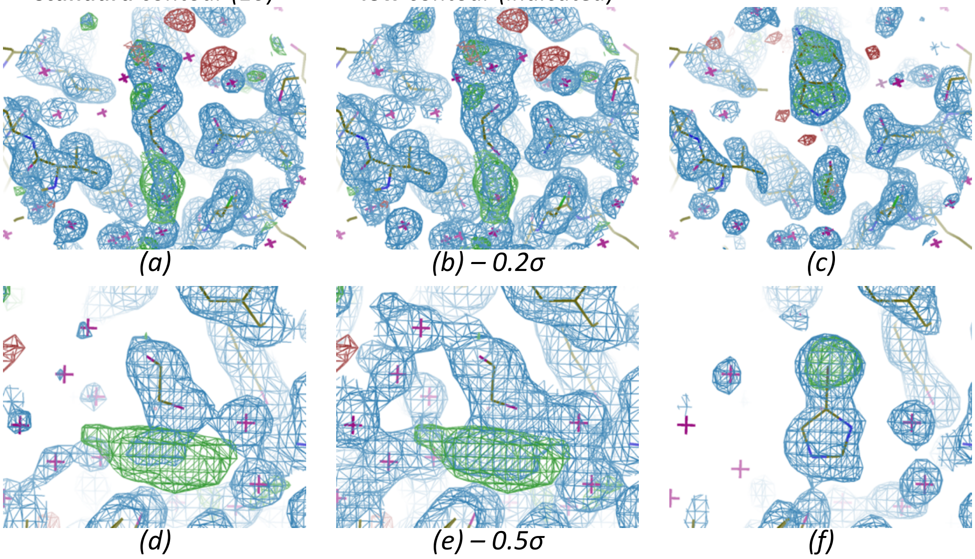

Supplementary Fig. 3. BAZ2B ligand examples. *Map layout and contour levels are as in Supplementary Fig. 1.*
Dataset x434: PanDDA maps show clear density for the ligand as well as an ethylene glycol that displaces a conserved binding site water.
Dataset x538: PanDDA maps show clear density that does not however match the shape of the labelled ligand; the clarity of the event map enabled the identification of the modelled ligand by its chemical connectivity, and the presence of a bromine, subsequently confirmed by calculation of an anomalous difference map (Supplementary Fig. 4).

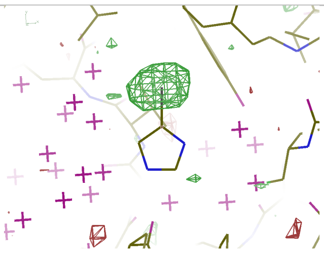

Supplementary Fig. 4. Anomalous difference map for dataset BAZ2B-x538. *The anomalous difference map (green/red; contoured at ±3σ) confirms the presence of the Bromine in dataset BAZ2B-x538. Data were collected at Diamond Light Source beamline i04-1.*

Standard maps, **standard** contour (1σ) | Standard maps, **low** contour (indicated) | PanDDA maps

Dataset: **x596**
Reso: 1.55Å
BDC: 0.86

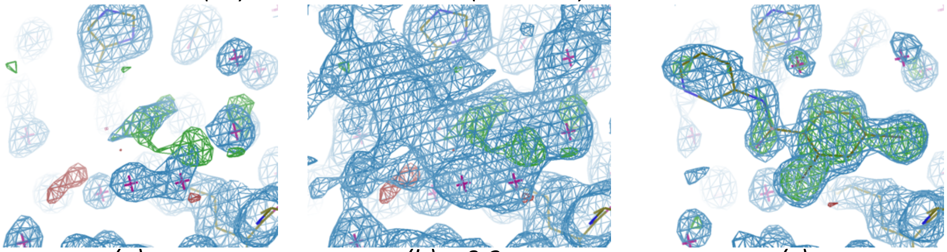

(a) (b) – 0.2σ (c)

Supplementary Fig. 5. SP100 ligand examples. *Map layout and contour levels are as in Supplementary Fig. 1.*
Dataset x596: Conventional maps show evidence for nothing but solvent. PanDDA maps show clear density for a weakly bound ligand.

Standard maps, **standard** contour (1σ) | Standard maps, **low** contour (indicated) | PanDDA maps

Dataset: **x050**
Reso: 1.52Å
BDC: 0.86

Dataset: **x062**
Reso: 1.67Å
BDC: 0.82

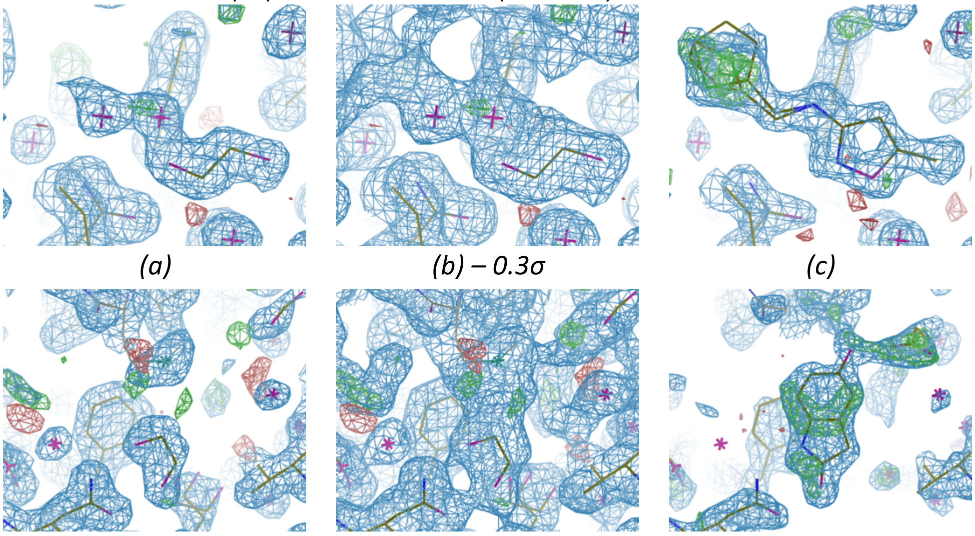

(a) (b) – 0.3σ (c)

(d) (e) – 0.1σ (f)

Supplementary Fig. 6. BRD1 ligand examples. *Map layout and contour levels are as in Supplementary Fig. 1.*
Dataset x050: Conventional maps show no density in the binding site that is not accounted for by the pre-existing ground-state solvent model. PanDDA maps show clear density for the ligand, and show that the solvent-exposed ring, which contains a sulphur, is discretely disordered, and present in two conformations.
Dataset x062: Conventional maps show poor and unconvincing evidence for a bound ligand. PanDDA maps show clear density for the ligand, and associated side-chain conformations. The ligand binding causes a strained conformation for a crystal symmetry methionine, potentially disturbing the crystal packing and explaining the imperfect event density.

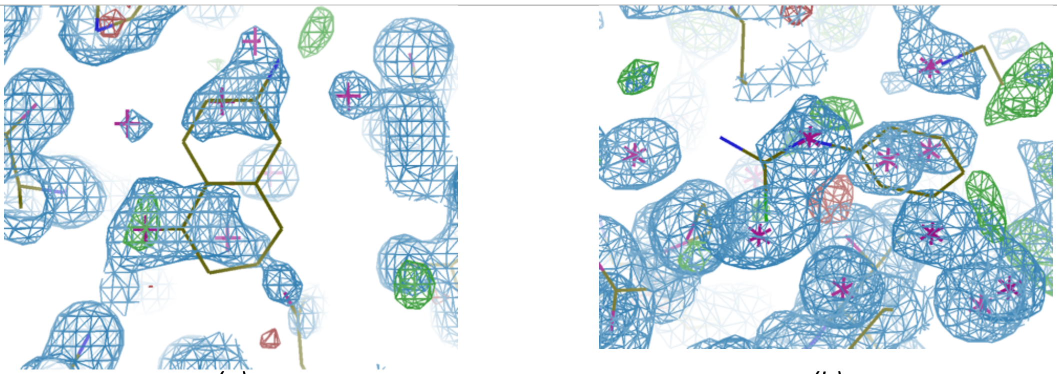

(a) (b)

Supplementary Fig. 7. Weak ligands can be rejected by conventional visual confirmation methods*. Final, refined $2mF_o$-DF_c (blue, 0.5σ) and mF_o-DF_c (green/red, ±3σ) for two ligands that are not validated by the conventional visual agreement between model and experimental density. (a) Ligand in dataset JMJD2D x443 (Supplementary Fig. 1g-i). (b) Ligand in dataset JMJD2D x542 (Supplementary Fig. 1m-o).*

Supplementary Table 1. JMJD2D ligand validation scores. *Rows are sorted by final refined occupancy. The residue column annotates different sites in the same crystal (label consists of the chain ID and residue number of the modelled ligand). The resolution limits of the data were automatically selected during data processing, typically where mean(I/sigma) falls below two. R-meas is given over the full range of the diffraction data. Other validation metrics are as defined in Methods. RMSDs marked by asterisks are large because ligands were manually remodelled after initial placement. RMSD is missing where ligands had atoms added or removed, e.g. through alternative conformations.*

Dataset	Residue	Res. (Å)	R-meas	Occ.	RSCC	RSZD	B Ratio	RMSD (Å)
x336	F 1	1.34	0.039	0.22	0.88	1.8	1.30	0.15
x401	F 1	1.48	0.025	0.22	0.87	1.7	1.45	0.12
x542	F 3	1.39	0.025	0.23	0.87	0.2	2.03	0.35
x543	F 1	1.40	0.027	0.25	0.87	0.0	2.30	0.42
x486	F 3	1.36	0.026	0.27	0.86	0.1	1.42	0.54
x443	F 2	1.34	0.028	0.37	0.91	2.2	2.54	0.22
x463	F 1	1.29	0.028	0.38	0.81	0.3	1.68	0.45
x365	F 1	1.25	0.025	0.39	0.88	1.2	1.33	0.16
x376	F 1	1.45	0.027	0.40	0.90	1.8	1.25	0.31
x396	F 1	1.40	0.023	0.41	0.91	0.7	1.70	
x443	F 1	1.34	0.028	0.41	0.77	1.7	1.88	0.20
x542	F 2	1.39	0.025	0.41	0.82	1.5	2.07	0.35
x396	F 2	1.40	0.023	0.43	0.91	0.5	1.43	
x486	F 2	1.36	0.026	0.43	0.83	0.5	2.07	0.32
x639	F 1	1.29	0.038	0.44	0.95	0.2	1.28	1.04*
x393	F 1	1.74	0.047	0.45	0.80	2.0	0.99	0.12
x486	F 1	1.36	0.026	0.46	0.93	2.3	1.19	0.20
x639	F 2	1.29	0.038	0.46	0.87	0.0	1.60	0.39
x637	F 1	1.40	0.026	0.47	0.94	2.1	1.50	0.19
x402	F 1	1.45	0.048	0.50	0.75	0.5	1.22	0.08
x555	F 1	1.60	0.027	0.50	0.91	0.6	1.54	0.54
x386	F 1	1.27	0.028	0.52	0.92	2.1	0.96	0.99
x486	F 4	1.36	0.026	0.52	0.87	1.0	1.91	0.21
x568	F 1	1.97	0.034	0.52	0.83	1.0	1.15	0.27
x623	F 1	1.14	0.053	0.53	0.91	0.0	1.63	0.16
x637	F 4	1.40	0.026	0.53	0.94	1.0	1.34	
x637	F 2	1.40	0.026	0.55	0.97	1.3	1.28	0.17
x637	F 3	1.40	0.026	0.55	0.98	0.8	0.95	1.07*
x494	F 1	1.35	0.029	0.56	0.94	0.1	1.08	0.14
x542	F 1	1.39	0.025	0.61	0.97	1.4	1.72	0.32
x611	F 1	1.15	0.051	0.61	0.87	0.7	2.15	0.30
x402	F 2	1.45	0.048	0.62	0.75	0.5	2.33	0.19
x620	F 1	1.25	0.036	0.62	0.78	0.2	2.49	0.23
x393	F 2	1.74	0.047	0.63	0.89	0.0	1.71	0.14
x395	F 1	1.43	0.040	0.66	0.91	1.1	0.72	0.13
x378	F 1	1.24	0.028	0.74	0.94	1.2	1.01	0.14
x392	F 1	1.35	0.025	0.98	1.00	0.0	0.86	0.10

Supplementary Table 2. BAZ2B ligand validation scores. *Details are as described in the legend to Supplementary Table 1. Dataset x538 reports the occupancy of all ligand atoms except the bromine, which is lower likely due to radiation damage.*

Dataset	Residue	Res. (Å)	R-meas	Occ.	RSCC	RSZD	B Ratio	RMSD (Å)
x538	C 1	1.77	0.028	0.40	0.96	1.7	1.05	0.27
x434	C 1	1.65	0.040	0.55	0.94	1.1	1.32	0.24
x559	C 1	1.78	0.027	0.55	0.91	1.4	1.14	0.30
x529	C 1	1.78	0.029	0.61	0.97	1.2	1.07	0.22
x492	C 1	1.78	0.040	0.64	0.97	1.8	1.16	0.17
x509	D 1	1.92	0.041	0.76	0.97	1.7	1.18	0.44
x481	C 1	1.65	0.040	0.77	0.95	0.9	1.26	0.16
x575	C 1	1.83	0.042	0.94	0.99	2.2	1.06	0.16
x583	C 1	1.80	0.038	0.94	0.98	1.1	1.21	0.33

Supplementary Table 3. SP100 ligand validation scores. *Details are as described in the legend to Supplementary Table 1.*

Dataset	Residue	Res. (Å)	R-meas	Occ.	RSCC	RSZD	B Ratio	RMSD (Å)
x601	F 1	1.57	0.056	0.38	0.82	0.3	1.26	4.99*
x596	F 2	1.55	0.053	0.40	0.86	1.4	1.66	0.43
x596	F 1	1.55	0.053	0.49	0.87	0.8	1.42	0.34

Supplementary Table 4. BRD1 ligand validation scores. *Details are as described in the legend to Supplementary Table 1. The bromine in the ligand in dataset x097 has a different occupancy to the rest of the ligand.*

Dataset	Residue	Res. (Å)	R-meas	Occ.	RSCC	RSZD	B Ratio	RMSD (Å)
x038	E 1	1.47	0.066	0.29	0.79	1.9	1.20	2.99*
x047	E 1	1.56	0.061	0.38	0.76	0.1	1.08	0.38
x099	E 1	1.52	0.048	0.45	0.76	1.2	1.42	0.56
x044	E 1	1.48	0.063	0.48	0.79	0.0	1.35	
x271	E 2	1.58	0.052	0.49	0.88	0.3	1.41	0.35
x069	E 1	1.49	0.062	0.55	0.79	0.1	1.41	0.37
x092	E 1	1.62	0.092	0.55	0.78	0.1	1.53	0.50
x082	E 2	1.50	0.069	0.57	0.86	0.1	1.23	
x062	E 1	1.67	0.067	0.58	0.95	0.7	1.15	0.20
x160	E 1	1.77	0.066	0.58	0.92	0.1	0.99	0.45
x081	E 1	1.61	0.064	0.59	0.86	1.3	1.41	0.48
x271	E 1	1.58	0.052	0.59	0.92	0.6	1.30	0.43
x225	E 1	1.56	0.068	0.60	0.88	2.6	1.16	0.42
x237	E 2	1.62	0.115	0.62	0.80	0.3	1.36	0.39
x099	E 3	1.52	0.048	0.63	0.89	0.1	1.47	
x223	E 1	1.62	0.149	0.63	0.91	1.8	1.03	0.22
x270	E 2	1.97	0.122	0.63	0.90	1.7	1.07	
x050	E 1	1.52	0.084	0.64	0.83	0.0	1.73	0.44
x099	E 2	1.52	0.048	0.66	0.90	0.2	1.41	
x334	E 1	1.61	0.072	0.67	0.91	0.4	1.24	0.31
x295	E 2	1.77	0.086	0.68	0.94	0.0	1.19	0.26
x083	E 2	1.50	0.050	0.69	0.91	0.1	1.30	0.29
x160	E 2	1.77	0.066	0.69	0.91	0.6	1.17	0.39
x295	E 1	1.77	0.086	0.70	0.91	0.1	1.57	0.41
x066	E 1	1.70	0.067	0.71	0.94	0.9	1.45	0.33
x225	E 2	1.56	0.068	0.71	0.93	0.1	1.52	0.91
x080	E 1	1.44	0.053	0.72	0.91	0.6	1.30	1.16*
x270	E 1	1.97	0.122	0.73	0.94	2.0	0.91	0.45
x261	E 1	1.58	0.056	0.74	0.89	1.0	1.17	0.23
x186	E 1	2.37	0.095	0.77	0.94	0.7	1.05	0.40

x049	E 1	1.46	0.049	0.78	0.96	1.6	1.18	0.15
x298	E 1	1.75	0.065	0.78	0.94	1.0	1.31	0.35
x274	E 2	1.75	0.095	0.80	0.95	0.2	1.21	0.21
x167	E 2	1.61	0.066	0.82	0.96	0.2	1.02	0.32
x310	E 1	1.76	0.210	0.84	0.93	1.9	0.98	0.40
x136	E 1	2.27	0.190	0.86	0.94	1.5	1.05	0.47
x084	E 2	2.12	0.141	0.87	0.96	0.9	1.17	0.27
x310	E 2	1.76	0.210	0.88	0.91	0.2	1.05	0.38
x186	E 2	2.37	0.095	0.89	0.95	1.0	1.32	0.49
x167	E 1	1.61	0.066	0.90	0.90	0.3	1.19	0.45
x274	E 1	1.75	0.095	0.91	0.96	1.7	0.99	0.15
x325	E 1	1.50	0.071	0.92	0.97	0.1	1.12	
x083	E 1	1.50	0.050	0.95	0.94	1.8	1.17	0.30
x084	E 1	2.12	0.141	0.95	0.96	2.1	0.99	0.40
x082	E 1	1.50	0.069	0.97	0.94	2.2	1.12	0.36
x093	E 1	1.78	0.086	0.97	0.97	1.3	0.98	0.14
x237	E 1	1.62	0.115	0.97	0.95	1.3	0.97	0.16
x284	E 1	1.43	0.047	0.98	0.95	0.1	1.37	0.25
x083	E 3	1.50	0.050	0.99	0.96	0.4	1.38	0.11
x249	E 1	1.52	0.067	0.99	0.95	0.5	1.21	1.23*
x093	E 2	1.78	0.086	1.00	0.97	1.1	1.16	0.11
x097	E 1	1.48	0.053	1.00	0.96	0.6	1.12	0.17
x098	E 1	1.56	0.085	1.00	0.93	2.1	1.77	0.16
x098	E 2	1.56	0.085	1.00	0.97	0.4	1.06	0.09
x136	E 2	2.27	0.190	1.00	0.92	1.2	1.15	1.10*
x223	E 2	1.62	0.149	1.00	0.96	2.0	1.30	0.16
x242	E 1	1.54	0.063	1.00	0.94	0.7	0.92	0.50
x242	E 2	1.54	0.063	1.00	0.96	0.8	1.22	0.22
x249	E 2	1.52	0.067	1.00	0.96	0.3	0.95	0.11
x258	E 1	1.50	0.065	1.00	0.93	0.3	1.27	0.30
x258	E 2	1.50	0.065	1.00	0.94	1.2	1.48	0.22
x261	E 2	1.58	0.056	1.00	0.95	0.3	1.33	0.24
x276	E 1	1.63	0.078	1.00	0.97	1.6	1.14	0.12
x292	E 1	1.57	0.053	1.00	0.97	0.3	1.00	
x292	E 2	1.57	0.053	1.00	0.97	0.1	0.97	

Supplementary Table 5. PDB Accession Numbers and Zenodo Links. *First row for each dataset: PDB codes for all modelled structures (as shown in Table 1). Second row for each dataset: PDB codes (shown as a range) for the dimple-refined structures that were not modelled (either were not detected to contain a ligand, contained a ligand bound in the crystal contacts or where the ligand could not be unambiguously identified). The third and fourth rows for each dataset respectively contain links to interactive HTML summary pages and links to repositories containing all crystallographic data and models as zip files for easy download.*

Dataset		
JMJD2D	PDB Codes (modelled/bound)	5PH0, 5PH1, 5PH2, 5PH3, 5PH4, 5PH5, 5PH6, 5PH7, 5PH8, 5PH9, 5PHA, 5PHB, 5PHC, 5PHD, 5PHE, 5PHF, 5PHG, 5PHH, 5PHI, 5PHJ, 5PHK, 5PHL, 5PHM, 5PHN
	PDB Codes (unmodelled/unbound)	5PHO - 5PNW
	Interactive summary	https://zenodo.org/record/290220/files/0_index.html
	Models & Data Zip Files	https://zenodo.org/record/48770
BAZ2B	PDB Codes (modelled/bound)	5PB7, 5PB8, 5PB9, 5PBA, 5PBB, 5PBC, 5PBD, 5PBE, 5PBF
	PDB Codes (unmodelled/unbound)	5PBG - 5PGT
	Interactive summary	https://zenodo.org/record/290199/files/0_index.html
	Models & Data Zip Files	https://zenodo.org/record/48768
SP100	PDB Codes (modelled/bound)	5PWC, 5PWD
	PDB Codes (unmodelled/unbound)	5PWE - 5PZJ
	Interactive summary	https://zenodo.org/record/290201/files/0_index.html
	Models & Data Zip Files	https://zenodo.org/record/48771
BRD1	PDB Codes (modelled/bound)	5PNX, 5PNY, 5PNZ, 5PO0, 5PO1, 5PO2, 5PO3, 5PO4, 5PO5, 5PO6, 5PO7, 5PO8, 5PO9, 5POA, 5POB, 5POC, 5POD, 5POE, 5POF, 5POG, 5POH, 5POI, 5POJ, 5POK, 5POL, 5POM, 5PON, 5POO, 5POP, 5POQ, 5POR, 5POS, 5POT, 5POU, 5POV, 5POW, 5POX, 5POY, 5POZ, 5PP0
	PDB Codes (unmodelled/unbound)	5PP1 - 5PWB
	Interactive summary	https://zenodo.org/record/290217/files/0_index.html
	Models & Data Zip Files	https://zenodo.org/record/48769

Supplementary Table 6. Crystal conditions and characteristics for each fragment screen. *R-free and R-work are calculated for the models after Dimple refinement but before model building.*

Protein	**JMJD2D**			**BAZ2B**		
Crystallisation Conditions	0.1M HEPES, pH 6.8-7.2,<br>0.2-0.25M ammonium sulphate,<br>25-29% PEG3350			0.1M MES, pH 6.0,<br>30% PEG600		
Cryo-Protectant (conc.)	Ethylene Glycol (25%)			Ethylene Glycol (25%)		
Solvent (conc.)	DMSO (10-30%)			Ethylene Glycol (25%)		
Soaking Time	50mins			1h		
Resolution Range (Å)	1.1-2.6			1.5-2.5		
Mean Resolution (SD) (Å)	1.45 (0.21)			1.79 (0.15)		
Space Group	P 43 21 2			C 2 2 21		
Mean Unit Cell Axes (Å)	71.42	82.17	96.57	58.03	71.42	150.41
Unit Cell Axes SD (%)	0.29 %	2.02 %	2.31 %	1.51 %	0.29 %	0.25 %
Unit Cell Volume SD (%)	0.80 %			3.03 %		
Median R-free / R-work	0.181 / 0.156			0.218 / 0.186		
NCBI Gene ID	55693			29994		
Domain Range	JmjN 18-64,<br>JmjC 182-295			1871-1955		
Domain Category	JmjN, JmjC (Jumonji)			Bromodomain		

Protein	**SP100**			**BRD1**		
Crystallisation Conditions	0.1M MES, pH 6.1,<br>20% PEG20K			0.1M bis-tris, pH 7.0,<br>30% PEG3350		
Cryo-Protectant (conc.)	Ethylene Glycol (30%)			Ethylene Glycol (30%)		
Solvent (conc.)	Ethylene Glycol (30%)			Ethylene Glycol (30%)		
Soaking Time	12h			2-4h		
Resolution Range (Å)	1.3-2.7			1.5-3.6		
Mean Resolution (SD) (Å)	1.72 (0.22)			1.76 (0.33)		
Space Group	C 1 2 1			P 21 21 21		
Mean Unit Cell Axes (Å)	127.67	45.39	83.36	55.46	56.42	101.76
Unit Cell Axes SD	0.09 %	0.21 %	0.20 %	0.75 %	0.30 %	0.27 %
Unit Cell Volume SD	0.50 %			1.05 %		
Median R-free / R-work	0.207 / 0.173			0.223 / 0.187		
NCBI Gene ID	6672			23774		
Domain Range	PHD 704-747,<br>Bromo 773-874			570-654		
Domain Category	PHD, Bromodomain			Bromodomain		

Supplementary Table 7. X-ray data and refinement statistics. *Data statistics for a representative ligand-bound structure for each of the fragment screens. Statistics were calculated for 20 resolution bins for all data. Statistics in brackets are shown for the highest resolution shell. Data statistics were calculated using phenix.table_one, except for BRD1, where merging statistics were calculated by aimless (rows extracted from aimless logfile indicated with *). Full X-ray data statistics for all datasets available on request.*

Dataset	**JMJD2D-x336**	**BAZ2B-x434**
Wavelength	0.9762	0.92
Resolution range	29.09 - 1.341 (1.364 - 1.341)	27.42 - 1.656 (1.684 - 1.656)
Space group	P 43 21 2	C 2 2 21
Unit cell	71.467 71.467 150.264<br>90 90 90	81.403 96.831 57.784<br>90 90 90
Total reflections	174416 (7761)	49801 (1684)
Unique reflections	87401 (4007)	25100 (889)
Multiplicity	2.0 (1.9)	2.0 (1.9)
Completeness (%)	99.52 (92.58)	90.98 (64.99)
Mean I/sigma(I)	15.60 (2.33)	14.46 (1.54)
Wilson B-factor	12.99	27.91
R-merge	0.02782 (0.3209)	0.02806 (0.4553)
R-meas	0.03935 (0.4539)	0.03968 (0.6439)
R-pim	0.02782 (0.3209)	0.02806 (0.4553)
CC1/2	0.998 (0.76)	0.998 (0.813)
CC*	1 (0.929)	1 (0.947)
Reflections used in refinement	87400 (4007)	25097 (889)
Reflections used for R-free	4299 (193)	1257 (37)
R-work	0.1292 (0.1857)	0.1777 (0.3511)
R-free	0.1626 (0.2095)	0.2033 (0.4177)
CC(work)	0.974 (0.866)	0.950 (0.411)
CC(free)	0.964 (0.850)	0.938 (0.037)
Number of non-hydrogen atoms	3476	1231
macromolecules	2937	998
ligands	79	26
solvent	460	207
Protein residues	331	115
RMS(bonds)	0.006	0.007
RMS(angles)	1.1	1.01
Ramachandran favored (%)	98.46	99.12
Ramachandran allowed (%)	1.23	0.88
Ramachandran outliers (%)	0.31	0
Rotamer outliers (%)	1.92	1.77
Clashscore	3.88	3.94
Average B-factor	19.84	34.13
macromolecules	16.97	31.22
ligands	28.77	38.69
solvent	36.6	47.62

Dataset	SP100-x596	BRD1-x038
Wavelength	0.92	0.92
Resolution range	27.77 - 1.549 (1.576 - 1.549)	21.45 - 1.47 (1.495 - 1.47)
Space group	C 1 2 1	P 21 21 21
Unit cell	127.74 45.415 83.292 90 102.233 90	55.4126 56.4243 101.725 90 90 90
Total reflections	130074 (6264)	*319382 (39429)
Unique reflections	66714 (3254)	54845 (2726)
Multiplicity	1.9 (1.9)	*5.8 (5.9)
Completeness (%)	97.76 (95.57)	99.64 (99.85)
Mean I/sigma(I)	12.16 (1.64)	*14.3 (2.9)
Wilson B-factor	20.01	17.38
R-merge	0.03724 (0.4599)	*0.055 (0.516)
R-meas	0.05267 (0.6504)	*0.066 (0.626)
R-pim	0.03724 (0.4599)	*0.037 (0.350)
CC1/2	0.998 (0.599)	*0.998 (0.795)
CC*	1 (0.866)	-
Reflections used in refinement	66710 (3254)	54842 (2726)
Reflections used for R-free	3333 (156)	2673 (133)
R-work	0.1810 (0.2785)	0.1890 (0.2781)
R-free	0.2099 (0.3316)	0.2118 (0.2950)
CC(work)	0.964 (0.708)	-
CC(free)	0.954 (0.672)	-
Number of non-hydrogen atoms	3511	2375
macromolecules	2936	2082
ligands	63	24
solvent	512	269
Protein residues	340	246
RMS(bonds)	0.012	0.006
RMS(angles)	1.23	0.93
Ramachandran favored (%)	98.48	100
Ramachandran allowed (%)	1.52	0
Ramachandran outliers (%)	0	0
Rotamer outliers (%)	1.47	0.9
Clashscore	4.92	2.63
Average B-factor	28.19	23.05
macromolecules	26.09	22.06
ligands	36.21	24.79
solvent	39.24	30.58

Supplementary Note 1 Fragment Screening Datasets

Each fragment screening dataset is comprised of datasets collected on crystals where one different compound was added to each crystal. Proteins and crystallization conditions are listed in Supplementary Table 6. Crystals were frozen in liquid nitrogen after compound soaking; the full experimental details vary considerably, even within some experiments, and will be published elsewhere. All data were collected on beamline I04-1 of Diamond Light Source. X-ray statistics for a representative dataset from each fragment screen are shown in Supplementary Table 7.

Lysine-specific demethylase 4D (JMJD2D) is a lysine-demethylase from the KDM4 family. This crystal form was soaked in the presence of the co-factor analogue N-oxalylglycine (NOG) tightly bound to the metal in the main binding site. Consistently, refinement of ground-state crystals indicates both metal and NOG are not present at full occupancy, and one fragment evidently exploits this by binding to empty sites in the crystal; this low-occupancy event is clearly revealed by PanDDA (main text; Figure 3).

The PanDDA analysis identified multiple allosteric binders that were missed by conventional inspection (main text; Figure 4d). Several fragments induce the reordering of an alpha helix at the C-terminal of the protein (Figure 4a-c, Supplementary Fig. 1a-c). Conventional density maps did not permit these complex reordering to be modelled (Figure 4a, Supplementary Fig. 1a), but this was simple in the PanDDA event maps, as they show only the density for the new conformation (Figure 4b, Supplementary Fig. 1c).

Compound dispensing and dataset labelling errors introduced through the complexity of exploratory fragment screening experiments, and possible cross-contamination in the compound library, provide a natural experiment for blinded ligand identification. For several strong hits from the BAZ2B, JMJD2D and BRD1 screens, the density did not allow fitting of the originally assigned ligand. However, unlike conventional maps, the PanDDA event maps enabled unambiguous reassignment of the correct compound, provided its chemical connectivity was unique within the library (Supplementary Fig. 1d-f, Supplementary Fig. 3d-f); anomalous difference maps can be used to confirm the modelling of some ligands (Supplementary Fig. 4).

When no fragments were seen to bind strongly, an objective measure of significance was vital to rationalise whether to attempt modelling into weak density. For a fragment screen against nuclear auto-antigen SP-100 (SP100), there were no clear hits from the initial analysis. Tentative models could be built into conventional maps for three datasets, even if all were subjectively assigned low confidence. In contrast, PanDDA Z-maps objectively clarified this ambiguity since none of the datasets had statistically significant deviation from the ensemble density to support the models: a bound but unmodelled buffer molecule had yielded misleading peaks in conventional density. Instead, two binders were identified that could be confidently modelled (one of which is shown in Supplementary Fig. 5).

Bromodomain-containing protein 1 (BRD1) presented the challenge of modelling with a high screening hit rate. Initial manual inspection yielded 29 binders, whereas PanDDA analysis revealed a further 13 binders (two examples are shown in Supplementary Fig. 6), an additional binding site, and revealed mis-assigned compounds in two existing models. Binding of chemically elaborated fragment hits was also analysed more reliably by PanDDA than conventionally (8 and 3 binders respectively), using the ground state characterisation from the initial analysis.

Supplementary Note 2 Ligand Screening Studies

In a conventional ligand-binding study of 35 datasets with the same crystal form (1.5 – 2.5Å) of bromodomain of human CREB binding protein (CREBBP), PanDDA retrospectively enabled the modelling of 2 ligands previously discarded due to unconvincing conventional maps (one example shown in Supplementary Fig. 8), while confirming all previously identified ligands. CREBBP had previously been co-crystallised with putative ligands; in this analysis, ground state datasets were identified by iterative rejection of bound-state datasets, and the former then used to review the latter. The density is unambiguous, even though the experiment is non-ideal due to the low number of ground-state datasets, which leads to event density of lower quality than observed in the fragment screening datasets.

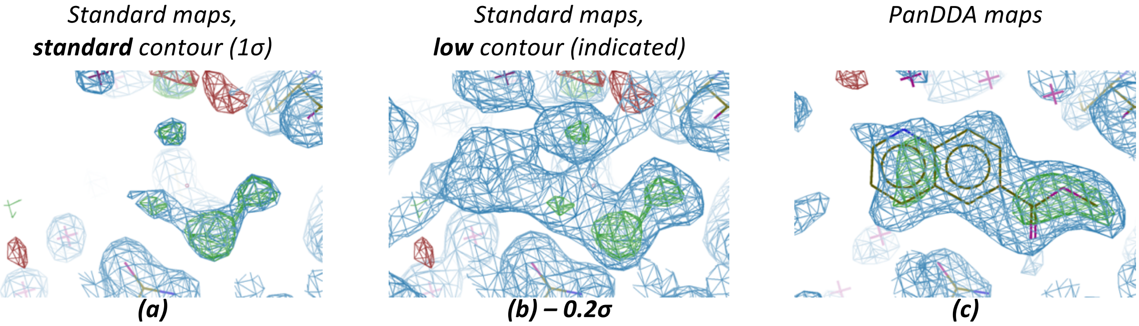

Supplementary Fig. 8. Weakly-bound ligands are identified in datasets from traditional ligand-screening studies. *(a)-(c) CREBBP co-crystallized ligand, 1.77Å dataset. Conventional maps in (a) and (b) (blue: 2mF_o–DF_c: green/red: mF_o–DF_c, ±3σ) show no convincing evidence for the ligand, even at low contour, while PanDDA maps in (c) (blue: event map, 1.5σ, BDC = 0.86; green/red: Z-map, ±3σ) show the ligand unambiguously; weaker density corresponds to the more solvent-exposed side of the ligand.*

Supplementary Methods

PanDDA Implementation

The PanDDA algorithm is implemented as shown in Supplementary Fig. 9.

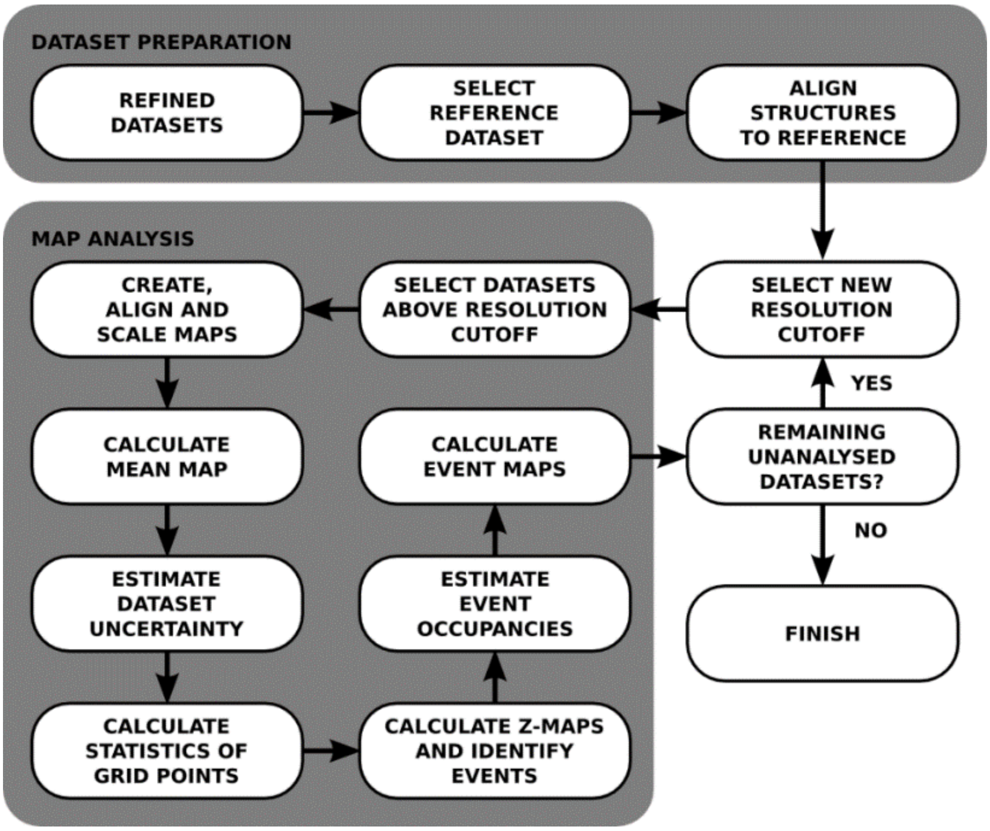

Supplementary Fig. 9. Top-level algorithm of the implemented PanDDA method. *The input to the PanDDA method is a series of refined datasets, which must contain similar structures. These structures are aligned, and these alignments are used to align the electron density maps for comparison. Datasets are analysed in resolution shells, with high resolution datasets being used multiple times to characterise lower-resolution shells. This iteration through resolution shells continues until there are no remaining unanalysed datasets.*

Flexible Alignment

The atomic composition of models to be aligned are required to be the same, so that they vary only in atomic coordinates, B-factors, and occupancies; i.e. the hierarchy of the model must be the same, but actual parameter values can vary. In practice, this is most easily achieved by using the same starting model for molecular replacement, followed only by refinement but no automated rebuilding.

An alignment is generated for each c-alpha by selecting the local environment of the c-alpha (backbone atoms of residues within 10Å), and calculating the least-squares fit of this selection to the corresponding atoms in the reference structure. After alignment, this process yields one rotation-translation matrix for each c-alpha of the protein, aligning the local environment of the c-alpha to the reference protein.

Coordinates are transformed between reference and native (crystallographic) frames as Voronoi cells of the c-alphas, where each point in space is transformed by the alignment associated with the nearest c-alpha (Supplementary Fig. 10).

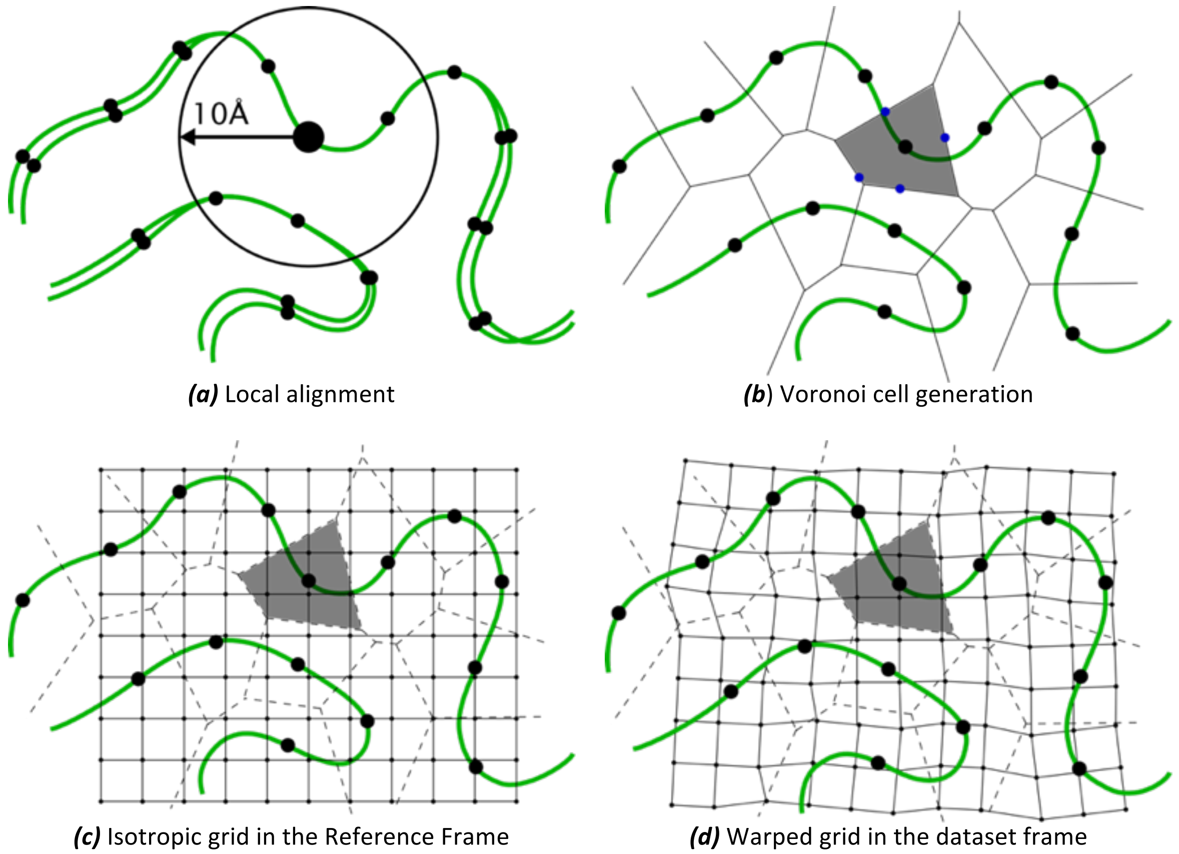

Supplementary Fig. 10. Alignment and transformation of crystallographic datasets. *(a) For each c-alpha in the structure, all residue atoms within 10Å are selected and aligned to the equivalent atoms in the reference structure. The resulting alignment transformation is assigned to that c-alpha. (b) Voronoi cells are produced in the reference structure for each of the c-alphas. Coordinates within the cells are transformed by the transformation matrix of the associated (closest) c-alpha. (c,d) To align the electron density maps, an isotropic grid is created in the reference frame. This is transformed to each dataset's frame using the corresponding alignment, creating a non-isotropic grid in the dataset frame.*

Uncertainty and Z-Map Calculation

The uncertainty parameter, σ_i, of each dataset is estimated by characterising the deviations from the mean map. Plotting the quantiles of the sorted differences from the mean map against the theoretical quantiles from a normal distribution (Supplementary Fig. 11a), the uncertainty of the dataset can be estimated as the slope of the central portion (between ±1.5 quantiles). Once the variability of each point, s_m, has been estimated, Z-scores are calculated as in (5). These Z-scores show increased normality when quantiles are plotted against those of a normal distribution (Supplementary Fig. 11b).

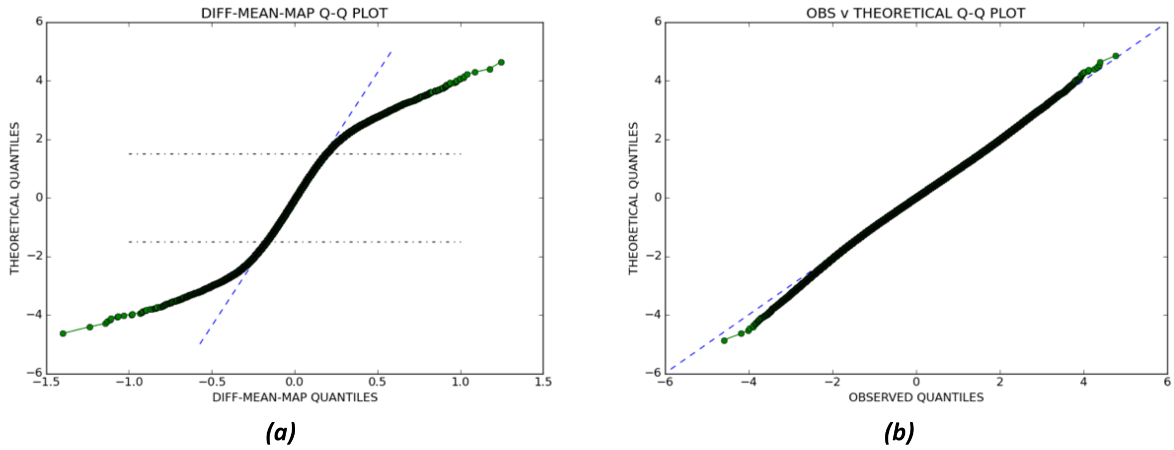

Supplementary Fig. 11. Characterisation of deviations from the mean-map. *(a) Example Q-Q plots against the normal distribution for the differences from the mean-map. (b) Example Q-Q plots against the normal distribution for the z-map. There is increased normality for the Z-map values compared to the simple difference from the mean map.*

Estimation of Density Variability

The estimation of the variability s_m at each point as observed throughout the whole set of datasets, requires the joint probability distribution function of the model in (3) (main text):

$$\mathrm{JPDF}_m \sim \left(\prod_{i=1}^{n} \frac{1}{\sqrt{\sigma_i^2 + s_m^2}} \right) * \exp\left(- \sum_{i=1}^{n} \frac{\left(\rho_{i,m}^{observed} - \mu_m\right)^2}{2\left(\sigma_i^2 + s_m^2\right)} \right). \tag{6}$$

Taking the logarithm, and maximising for s_m, yields

$$\sum_{i=1}^{n} \frac{\left(\rho_{i,m}^{observed} - \mu_m\right)^2}{\left(\sigma_i^2 + s_m^2\right)^2} - \sum_{i=1}^{n} \frac{1}{\left(\sigma_i^2 + s_m^2\right)} = 0. \tag{7}$$

Using the mean ground state map values, μ_m, experimental values, $\rho_{i,m}^{observed}$, and estimated uncertainties, σ_i, this equation can be numerically solved to find an estimate for s_m.

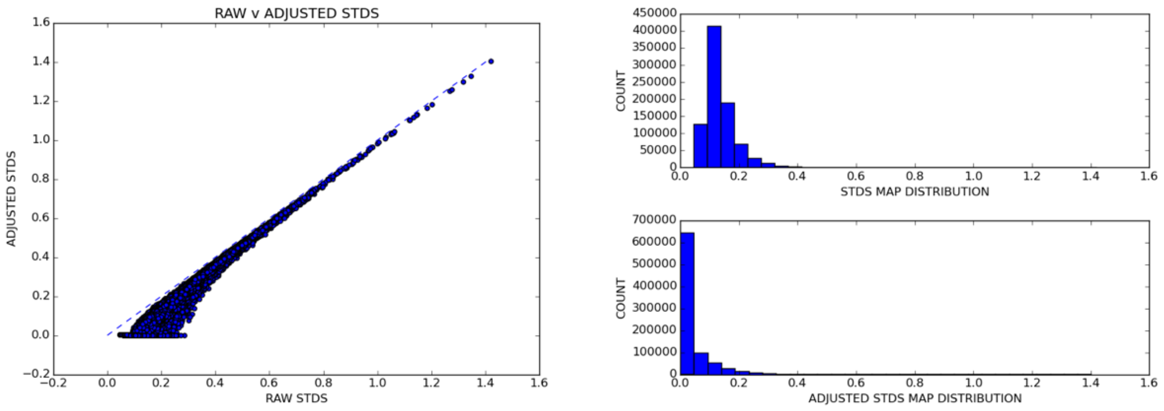

(a) Scatter plot of raw v adjusted s-values

(b) Histogram of raw v adjusted s-values

Supplementary Fig. 12. Calculation of s-Adjusted Map Values. *(a) Example "raw" variation of each grid point plotted against the "adjusted" variation of each grid point (after the uncertainty of each dataset has been accounted for. The "adjusted" value is decreased relative to the "raw" value, and for small "raw" values this variation is explained by the uncertainty in the datasets only. (b) Corresponding histograms of the "raw" and "adjusted" variation of the grid points. The "raw" histogram shows multiple grid points with variation between 0.05-0.40. However, the "adjusted" deviations show most variations are accounted for by experimental error, resulting in an "adjusted" value between 0-0.05.*

Statistical Model Convergence

The number of datasets required for the adjusted standard deviation s_m to converge statistically was analysed for 3 fragment screens, each of which had more than 100 empty datasets. The best estimate of s_m is assumed to be obtained by using all the datasets. To determine a convergence cutoff, available datasets were jack-knifed randomly into two halves and analysed with PanDDA.

The 90, 95, and 99% quantiles of the absolute difference between the derived s_m values for the jack-knifed halves define the 90, 95, and 99% convergence cutoffs, respectively. These derived cutoffs are recorded in Supplementary Table 8. Units are measured in sigma, as the analysis was performed on sigma-scaled maps. Multiple PanDDAs were then run with different numbers of randomly-selected datasets, and the percentage of s_m values within the 90, 95, and 99% convergence cutoffs of the best estimate (all datasets) was recorded. This was performed 5 times, with different seeds. The results of this convergence are shown in Supplementary Fig. 13.

Approximately 30 datasets were needed to reach the required cutoff, regardless of whether the 90, 95 or 99% cutoff was selected, as expected, implying this is the minimum number of datasets that should be used. It should be noted that this is the required number of datasets to achieve a convergence of s_m, which is a suppressive parameter; using fewer datasets are only introduces more noise, rather than losing signal. However, the fact that 30 datasets are required to yield a complete description of the density is evidence that a considerable amount of variation is present between crystals.

Supplementary Table 8. S-Adjusted Map Convergence. *The JARID1B dataset is not further discussed in this paper.*

Protein	# Datasets	90% Cutoff (σ)	95% Cutoff (σ)	99% Cutoff (σ)
BAZ2B	170	0.05	0.07	0.10
JARID1B	120	0.03	0.04	0.07
JMJD2D	200	0.04	0.05	0.07

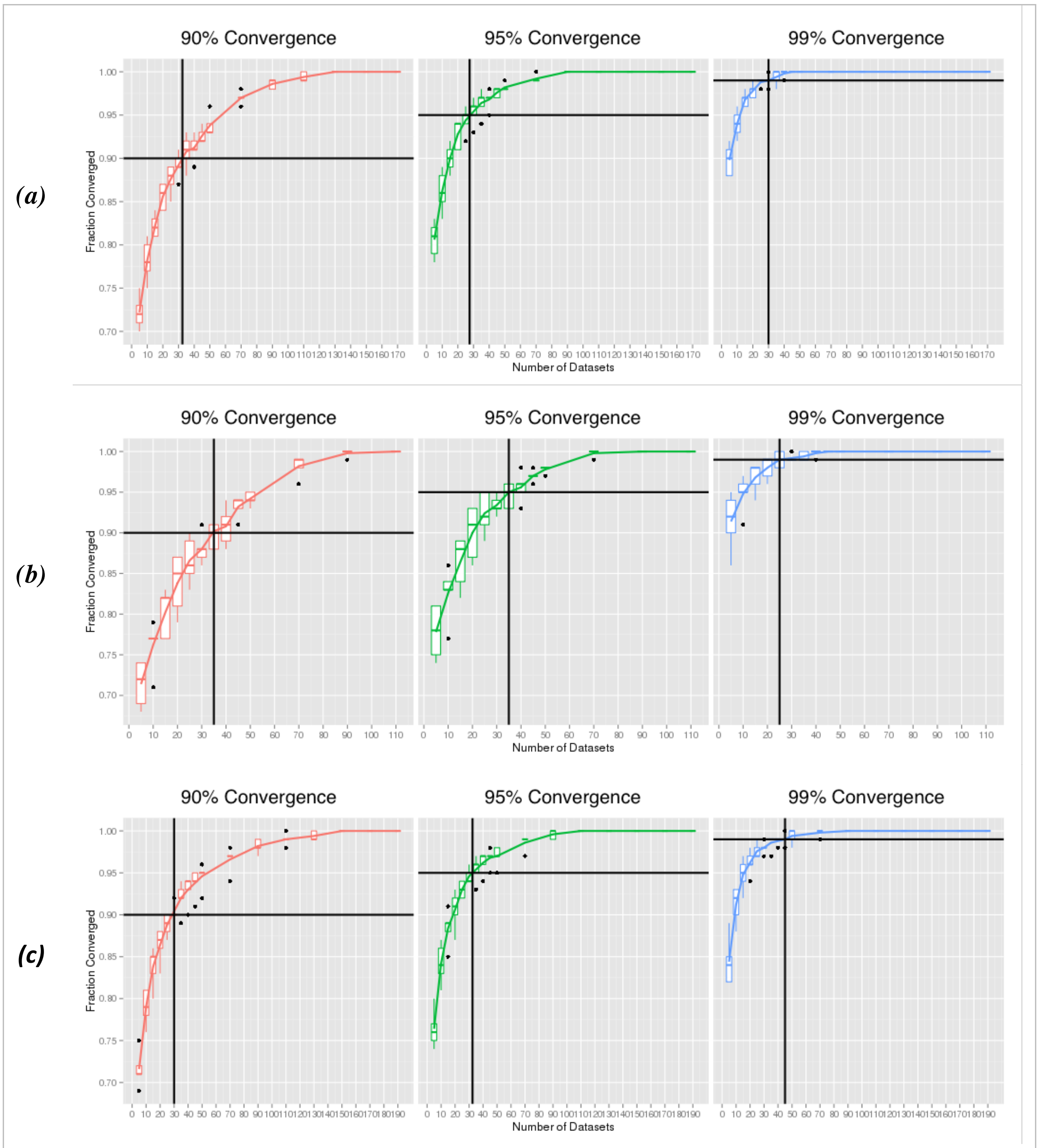

Supplementary Fig. 13. Convergence of adjusted s-map values. *(a) BAZ2B. (b) JARID1B (data not otherwise included). (c) JMJD2D.*

Background Density Correction

An example series of event maps for varying values of BDC is shown in Supplementary Fig. 14. The full $2mF_o$-DF_c map does not show clear density for the ligand (maps are shown at a low contour level of 0.4σ). At the appropriate value of BDC, the ligand density is clearly revealed, as is the density for the associated conformation of the protein.

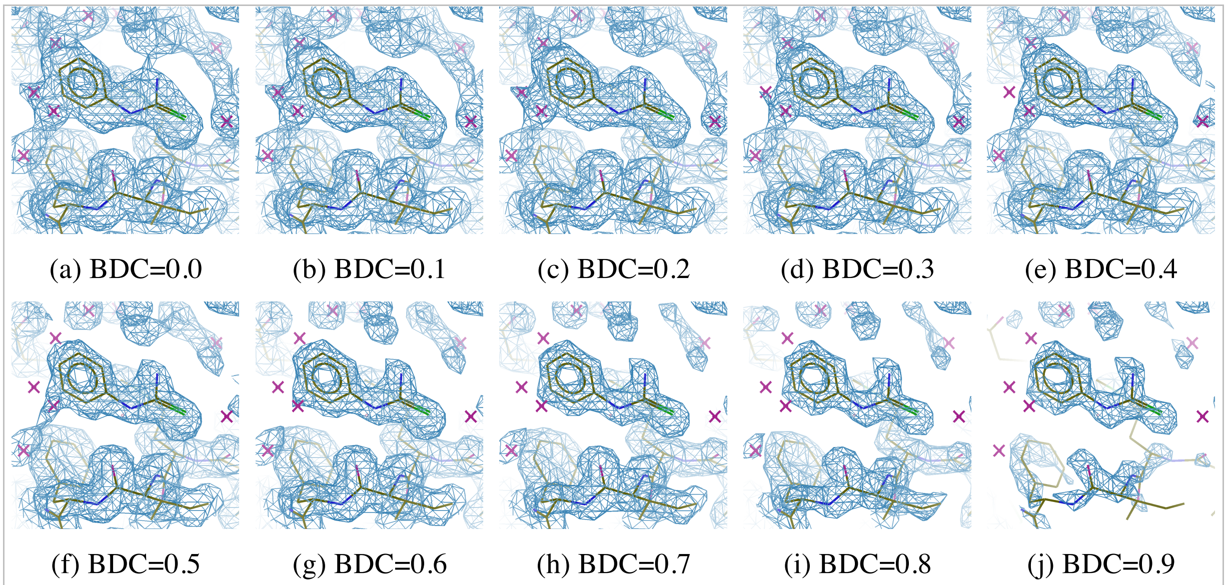

Supplementary Fig. 14. Example of the varying values of the background density correction factor. *The ligand shown is chain F, residue 1 from dataset JMJD2D-x542 (1.39Å). Maps are from refinement of the reference structure with Dimple where no ligand is modelled. All event maps are contoured at the same electron density value (equivalent to 0.4σ in the standard* $2mF_o$-DF_c *map which is the same as the BDC=0.0 map). As BDC increases, the density for water molecules from the ground state (shown superposed with the modelled ligand) is removed and the density for the ligand becomes clear. The automatically estimated value of BDC is 0.88.*

The background density correction is estimated automatically by maximising the difference between the local correlation and the global correlation of a background-corrected map to the ground state, as described in Methods. An example of this is shown in Supplementary Fig. 15.

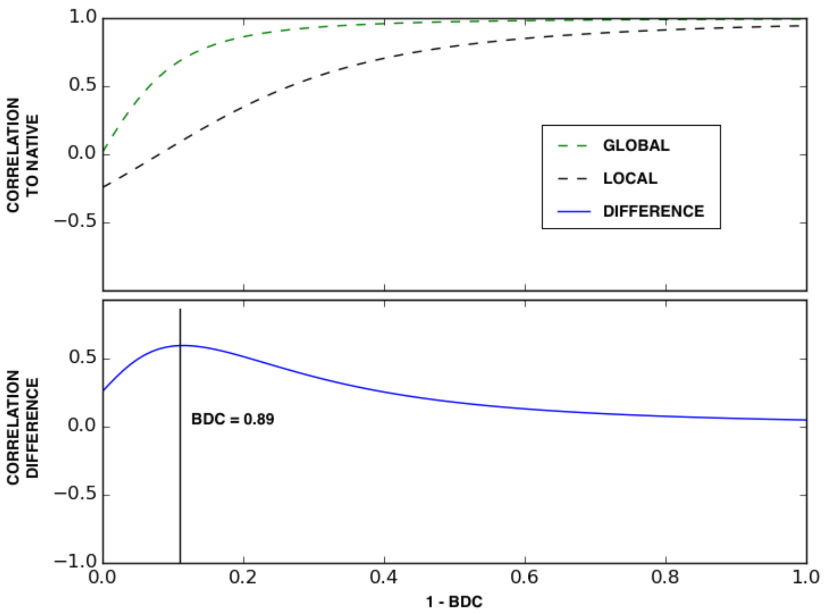

Supplementary Fig. 15. Example of how background density correction is estimated. *Global (green, dashed) and local (black, dashed) correlations between the ground state and background-corrected maps for different values of BDC. The local correlation is calculated over the voxels identified by the Z-map, expanded by 1Å; the global correlation uses all voxels in the map within 1.8Å of any protein atom. The BDC is estimated as the value that maximises the difference between local and global correlation; in this example, it is 0.89.*

BDC is chosen only to maximise contrast between the ground-state map and the event map. We note that an artefact is that density that has not changed (e.g. surrounding protein) appears in corrected maps at somewhat lower levels than density that has changed. However, such relative changes in signal are not problematic for model building in practice, and moreover, the discrepancy decreases for weaker changed-states, where the event map is most useful.

Since BDC is the fraction of ground-state map to be subtracted, the remainder (1-BDC) is the fraction of map representing the changed state, and therefore related to occupancy. The relation is shown in Supplementary Fig. 16: 1-BDC is consistently approximately half of the refined occupancy (fitted slope of 2.2), even for fully occupied ligands. This difference is likely partially due to "phase absence" in event maps, which is corrected only once the change is modelled and refined. Specifically, the amount of changed-state density present in the uncorrected map will be lower than in the final refined maps, as the former is calculated with phases derived from a model that does not yet include the changed state.

This relationship between BDC and occupancy provides real evidence for a direct influence of the local model phases on the local density. These phase effects likely also explain uneven density for changes (ligands): for map voxels where the changed state overlaps with atoms already modelled for the ground state, the modelled atoms more closely represent the actual electron density than where no atoms are modelled, leading to stronger density.

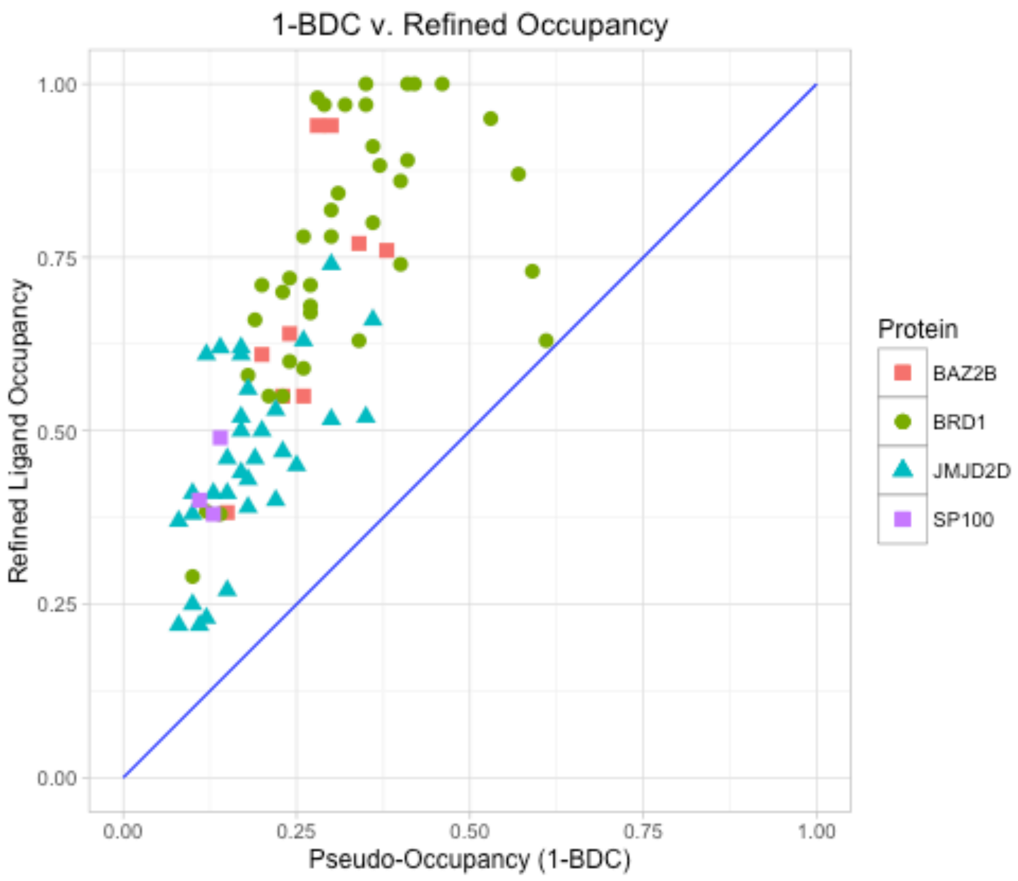

Supplementary Fig. 16. Relation between BDC and refined occupancy indicates effect of local model on phases. *Values are plotted for hits from four sets of experiments; refined occupancy is consistently larger than 1-BDC.*
