# Supplementary material for: A multi-crystal method for extracting obscured crystallographic states from conventionally uninterpretable electron density
Source: Nat Commun. 2017 Apr 24;8:15123. doi: 10.1038/ncomms15123 (PMC5413968; doi:10.1038/ncomms15123)
Supplement: Peer Review File [file ncomms15123-s2.pdf]

**Editorial Note:** This manuscript has been previously reviewed at another journal that is not operating a transparent peer review scheme. This document only contains reviewer comments and rebuttal letters for versions considered at Nature Communications.

**REVIEWERS' COMMENTS:**

**Reviewer #2 (Remarks to the Author):**

This is a very interesting manuscript that describes a combination of computational and experimental methods to recover weaker electron density for sites of heterogeneity in crystallographic maps. The work is thorough and presents several examples where the method shows an improvement over standard methods currently in use. The manuscript is well written and should be readily understood by those familiar with the interpretation of crystallographic density maps.
